# Elucidate multidimensionality of type 1 diabetes mellitus heterogeneity by multifaceted information

**DOI:** 10.1038/s41598-021-00388-2

**Published:** 2021-10-25

**Authors:** Shaw-Ji Chen, Jen-Liang Cheng, Sheng-An Lee, Tse-Yi Wang, Jyy-Yu Jang, Kuang-Chi Chen

**Affiliations:** 1 Department of Psychiatry, Mackay Memorial Hospital, Taitung, Taiwan; 2 Department of Medicine, Mackay Medical College, New Taipei City, Taiwan; 3 Department of Medical Informatics, Tzu Chi University, No. 701, Sec. 3, Zhongyang Rd., Hualien, 97004 Taiwan; 4 Department of Health Industry Management, Kainan University, Taoyuan, Taiwan

**Keywords:** Computational biology and bioinformatics, Systems biology

## Abstract

Type 1 diabetes (T1D) is an autoimmune disease. Different factors, including genetics and viruses may contribute to T1D, but the causes of T1D are not fully known, and there is currently no cure. The advent of high-throughput technologies has revolutionized the field of medicine and biology, and analysis of multi-source data along with clinical information has brought a better understanding of the mechanisms behind disease pathogenesis. The aim of this work was the development of a data repository linking clinical information and interactome studies in T1D. To address this goal, we analyzed the electronic health records and online databases of genes, proteins, miRNAs, and pathways to have a global view of T1D. There were common comorbid diseases such as anemia, hypertension, vitreous diseases, renal diseases, and atherosclerosis in the phenotypic disease networks. In the protein–protein interaction network, CASP3 and TNF were date-hub proteins involved in several pathways. Moreover, CTNNB1, IGF1R, and STAT3 were hub proteins, whereas miR-155-5p, miR-34a-5p, miR-23-3p, and miR-20a-5p were hub miRNAs in the gene-miRNA interaction network. Multiple levels of information including genetic, protein, miRNA and clinical data resulted in multiple results, which suggests the complementarity of multiple sources. With the integration of multifaceted information, it will shed light on the mechanisms underlying T1D; the provided data and repository has utility in understanding phenotypic disease networks for the potential development of comorbidities in T1D patients as well as the clues for further research on T1D comorbidities.

## Introduction

Diabetes mellitus (DM) is a group of metabolic diseases that involve the problems with the regulation of glucose in blood^1^. With diabetes, the body either makes insufficient insulin or defectively uses the insulin, resulting in hyperglycemia. Diabetes is divided into type 1 diabetes (TID), type 2 diabetes (T2D), and others (gestational diabetes mellitus and prediabetes). T2D is a complex endocrine and metabolic disease; T1D is an autoimmune disease. The insulin-producing pancreatic β-cells of T1D patients are destroyed by T lymphocytes and macrophages, so β-cells cannot produce sufficient insulin. The DM patients would easily develop unstable glucose levels with life threatening conditions of hypoglycemia (low blood sugar) or hyperglycemia (high blood sugar).

In the past, many studies on DM disorders (T1D, T2D, and others) only focused on specific pathways or mechanisms involving DM formulation, whereas a few studies explored the global picture of DM^2–5^. The emerging concept of “network medicine” assumes that the biological systems, like social or technological systems, are governed by simple and quantifiable organizational rules, albeit with lots of components and complicated relationships^6,7^. With the wealth of the interaction data, network biology provides a suitable framework to describe the cellular processes, quantify the perturbation patterns, and understand how their collective perturbations from subcellular networks affect disease states^8^. Moreover, the advent of high-throughput technologies have allowed the systematic and comprehensive mapping of the interactions between the biochemical entities that together represent the human interactome network. Access to high-throughput data has revolutionized the field of medicine and biology, and analysis of multi-omics data along with clinical information has brought a better understanding of the mechanisms behind disease pathogenesis^9^.

Hidalgo et al. constructed the phenotypic disease network (PDN) from medical records of 13 million elderly American inpatients to elucidate the comorbidity correlations between diseases and the disease progression preferentially along the links of the PDN^10^. Klimek et al. quantified diabetes comorbidity risks across life and gender using nationwide claims of two million Austrian inpatients^11^. Because all biochemical processes are governed by the proteins, the protein–protein interactions (PPIs) especially the proteins encoded by the casual and susceptible genes play crucial roles in orchestrating the regulatory variation^12–14^. The large-scale protein interaction networks enhance the knowledge about the molecular etiology of diseases and the discovery of putative protein targets with therapeutic significance^15,16^. Furthermore, microRNAs (miRNAs) are small, single-stranded, noncoding RNA molecules that negatively regulate gene by binding to the 3’ UTRs (untranslated region) of their target messenger RNAs (mRNAs), leading to mRNA degradation and protein target suppression^17^. Growing evidence indicates that miRNAs are very important in the regulation of physiological and pathological processes^18^, and some miRNAs might be used as potential biomarkers for cancer, cardiovascular disease, metabolic diseases and autoimmune diseases^19^. It is a challenge to elucidate multidimensionality of DM heterogeneity by multifaceted information^5^. The relation inference of network medicine is addressed by heterogeneous interconnected dynamics^7^.

The analysis of genotype–phenotype associations at multiple scales can provide a comprehensive view of novel insights into the cause and effect of diseases, and lead to a surprising interest in uncovering the organizing principles that govern the topology and the dynamics of various complex networks^8,20^. It is an application of network science that offers a suitable framework to describe global relationships between human disorders, associated genes and interactome networks. For diabetes, to better understand the disease conditions, it is necessary to analyze multifaceted information^5^. The aim of this work was the development of a data repository linking clinical data and interactome studies in T1D. We analyzed the electronic health records (EHRs) to construct the PDNs and find out comorbid diseases of T1D. Moreover, the PPI network, miRNA information, GO enrichment analysis^21^, and KEGG pathways^22,23^ were employed to have a global view of T1D.

## Material and methods

### Electronic health records

We used the EHRs from Taiwan National Health Insurance Research Database (NHIRD) in the study. The data gives a nation-wide picture of the medical condition of 99% of 23.78 million Taiwaneses. We analyzed the 2002–2008 hospitalization data and there were a total of 20,603,462 inpatient claims, pertaining to 8,044,512 persons (data approval number: NHIRD-104-293). Each record consists of the birthdate, gender, the date of visit, a main diagnosis and up to 4 side diagnoses, all specified by the International Classification of Diseases, Ninth Revision, Clinical Modification (ICD-9-CM) codes of up to 5 digits. Our hospitalization data consists of 759,683 diabetic inpatients who were diagnosed with 250.×× (T1D by 250.×1 or 250.×3, and T2D by 250.×0 or 250.×2) during 2002–2008 and stayed one or more nights in hospitals for DM treatment. The non-DM inpatients were considered as control inpatients. The numbers and proportions of these DM inpatients across ages and types are listed in Table 1. These data are consistent with the fact that T1D is usually diagnosed before 20 years old, and T2D is usually diagnosed after 40 years old^24^.

**Table 1:** The numbers and percentages of DM inpatients from 2002 to 2008.

Age group	T1D	T2D	Others	Total
[1, 10)	822 (80.75%)	173 (16.99%)	23 (2.26%)	1018 (100%)
[10, 20)	1912 (59.34%)	1262 (39.17%)	48 (1.49%)	3222 (100%)
[20, 30)	2088 (23.81%)	6034 (68.80%)	648 (7.39%)	8770 (100%)
[30, 40)	1746 (6.87%)	22,636 (89.07%)	1032 (4.06%)	25,414 (100%)
[40, 50)	1377 (1.88%)	71,492 (97.37%)	552 (0.75%)	73,421 (100%)
[50, 60)	1214 (0.82%)	145,947 (98.89%)	424 (0.29%)	147,585 (100%)
[60, 70)	1226 (0.65%)	185,581 (99.03%)	592 (0.32%)	187,399 (100%)
[70, 80)	1433 (0.69%)	207,022 (98.98%)	701 (0.34%)	209,156 (100%)
[80, 90)	611 (0.65%)	92,910 (99.04%)	290 (0.31%)	93,811 (100%)
90+	61 (0.62%)	9798 (99.10%)	28 (0.28%)	9887 (100%)
Total	12,490 (1.64%)	742,855 (97.78%)	4338 (0.57%)	759,683 (100%)

### Phenotypic disease network

The phenotypic disease network (PDN) is constructed in the form of a network with diseases as the nodes and pairwise comorbidity correlations between diseases as the links, and it can be viewed as a map of the progression of diseases^10^. The comorbidity correlation represents the "distance" between a pair of diseases. We employed the *ϕ*-correlation, a contingency coefficient, to quantify the comorbidity correlations of T1D inpatients^10^

$$ \phi_{ij} = (N \, N_{ij} - N_{i} N_{j} ){/}[N_{i} N_{j} {(}N{-}N_{i} {)(}N{-}N_{j} {)}]^{{0.{5}}} $$

where *N* is the total number of patients in the population, *N*_*i*_ and *N*_*j*_ are the prevalence of diseases *i* and *j*, and *N*_*ij*_ is the number of patients affected by both diseases. The top 0.6% of comorbidity links and their related nodes were selected in each PDN. In the PDN, we divided disease codes into 13 categories (Table S1), and excluded symptoms, injures, poisonings, pregnancies, external causes, and factors of morbidity. To evaluate the significance of each disease category, we used two-sample proportion test to compare whether the proportion of each disease category in one PDN was significantly different from the proportion of the same disease category in another PDN.

### Candidate genes associated with T1D

We searched the OMIM database^25^ with the keywords "DIABETES MELLITUS, INSULIN-DEPENDENT" or IDDM or "EARLY-ONSET DIABETES MELLITUS" or "JUVENILE-ONSET DIABETES" or "DIABETES MELLITUS TYPE 1", and collected 48 non-redundant candidate genes associated with T1D (Table S2). These genes were involved in the biological process of carbohydrate homeostasis and glucose homeostasis according to the GO^21^ annotation (the details listed in the Table S3), and constituted the seed proteins for constructing the following PPI network of T1D.

### Protein–protein interactions network

Protein–protein interactions (PPIs) are important because biological processes in human bodies are directly controlled in the level of protein proceedings^12^. Connecting the topological properties with biological knowledge will provide us more comprehensive information to understand the biological mechanisms. We aimed to analyze the contribution of the proteins encoding by T1D related genes to the pathogenesis of T1D and discover other key proteins cooperating with them by topological analyses. The candidate genes were converted into the seed proteins to obtain the PPI network from the STRING v.11.0 database^26^, a precomputed database for the exploration of PPIs. Given a list of the proteins as inputs, STRING will search for their neighbor interactors, and generate the PPI network consisting of these proteins and the interactions between them. We constructed the PPI network of T1D under the setting with 0.93 confidence based on active interaction sources from high-throughput lab experiments and previous knowledge in curated databases.

### Topological analysis of the PPI network

The topological analyses have been applied extensively in recent years. Degree centrality (DC), betweenness centrality (BC), and closeness centrality (CC) are widely adopted to evaluate nodes in a network^8^. The degree is the count of the direct links of a node in the network. The betweenness is the proportion of the number of shortest paths passing through a node to the number of all shortest paths in the network. The closeness is defined as the reciprocal average length of the shortest paths between a node and all other nodes. In a PPI network a node with high degree is considered as a hub protein, whereas a node with high betweenness is viewed as a bottleneck protein^8,13,27^. A node with high closeness is close to the other nodes in the network.

Furthermore, average degree (<*k*>), diameter (D), mean shortest path length (mspl), and the average clustering coefficient (acc) are viewed as global topological measurements of networks. Average degree (<*k*>) is the mean of all degree measurements of nodes in a network. Diameter (D) is the largest among all shortest paths in a network. The mean shortest path length (mspl) is calculated by averaging over all shortest paths between all pairs of nodes. The clustering coefficient is a measure of the local interconnectedness of the network. A network is considered as a small-world if it has a low mspl and a high acc^28^. In this study, the PPI network included a giant component and several small separate components derived from seed proteins. The structure of the PPI network was analyzed by Gephi v.0.9.2^29^, a program for analysis and visualization of very large networks.

### Retrieval of backbone from the PPI network

We aimed to identify the important proteins and the molecular connectivity between regulatory pathways related to T1D. In the PPI network the nodes with high BC are similar to heavily used intersections which have great influence on flow in the network^13,27^. Because most of the shortest paths in a network go through the high BC nodes, these nodes play as the bottlenecks which have more control over the network. The nodes with top 20% BC were considered as important and these proteins and the links between them were extracted from the giant component to constitute the backbone of the network.

### MiRNAs related to T1D

MiRNAs are related to immune system functions and β-cell metabolism, proliferation involving in T1D pathogenesis, and modulate mRNA expressions of the major T1D autoantigens^18,19^. A number of studies show that miRNAs have a vital role in the etiology and pathogenesis of DM and its complications^19,30,31^. Changes in miRNA expression levels in T1D are noticed because the dysregulation of miRNA expression might play important functions in moderating the development of T1D. We searched for miRNA of T1D from the HMDD v3.2 database^32^, a database collecting experiment-supported evidence for human miRNA and disease associations, and connected these miRNAs with our T1D backbone proteins to construct an interaction network.

### Ethical approval and consent for participation and publication

This study was approved and granted for exempt review by the Institutional Review Board (IRB) of Tzu Chi Hospital, Hualien, Taiwan, certificated by the Ministry of Health & Welfare, Taiwan (IRB approval number: IRB 104-114-C). All methods were carried out in accordance with relevant guidelines and regulations, and the informed consent was waived.

## Results

### EHR data and the PDN of T1D

**Figure 1:**
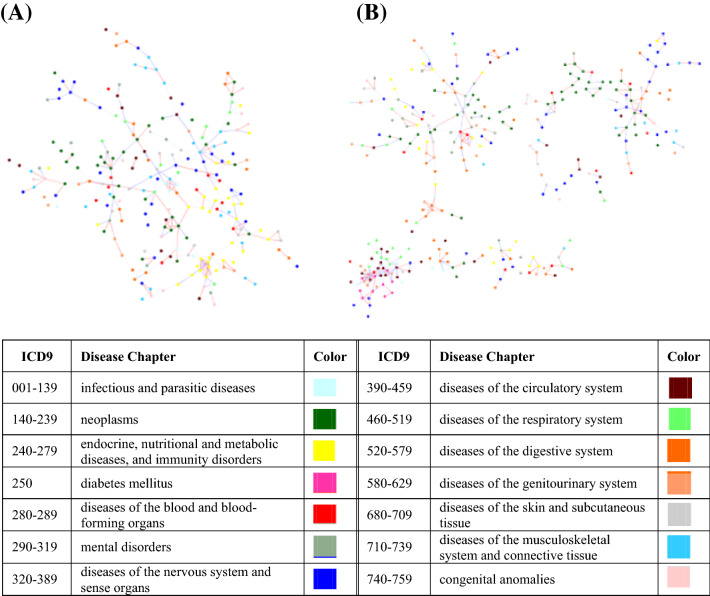
The PDNs of T1D for (**A**) Male and (**B**) Female inpatients. The PDN was generated using Gephi v0.9.2^29^ (https://gephi.org/).

We analyzed the hospitalization data in Taiwan from 2002 to 2008 to draw the PDNs of T1D consisting of 566 nodes with 644 links for male inpatients and 577 nodes with 667 links for female inpatients (Fig. 1). The PDNs of T1D were complex, mainly covering neoplasms (140–239), endocrine, nutritional and metabolic diseases, and immunity disorders (240–279), diseases of the nervous system and the sense organs (320–389), diseases of the circulatory system (390–459), and disease of the digestive system (520–579) for both male and female T1D inpatients (Table S4). Additionally, female T1D inpatients tended to have significantly more comorbidities than male T1D inpatients in diseases of the genitourinary system (580–629) by two-sample proportion test.

The PDNs of control group consisted of 722 nodes with 2358 links for male inpatients and 769 nodes with 2445 links for female inpatients (Figure S1). The PDNs of control group mainly covered neoplasms (140–239), diseases of the nervous system and the sense organs (320–389), diseases of the circulatory system (390–459), and disease of the digestive system (520–579) for both male and female control inpatients (Table S4). There was no different between male control inpatients and female control inpatients. When comparing the PDNs of TID inpatients with the PDNs of control inpatients, we found that the male TID inpatients had significantly more comorbidities than male control inpatients in diseases of the skin and subcutaneous tissue (680–709) and endocrine, nutritional and metabolic diseases (240–279). The male T1D inpatients had less comorbidities than male control inpatients in infectious and parasitic diseases (001–139).

Moreover, because T1D is usually diagnosed before the age of 20, we selected 1–20 T1D inpatients to construct the PDN (Figure S2(A), (B)). For male (or female) inpatients with T1D aged 1–20 years, the PDN has 323 (or 366) nodes and 741 (or 829) links. Both PDNs of male and female inpatients with T1D aged 1–20 years had more comorbid nodes in the same disease categories of 240–279, 320–389, 390–459, and 520–579 (Table S5). There was no different between young male T1D inpatients and young female T1D inpatients. For comparison, we also selected 40–60 T1D inpatients to construct the PDNs (Figure S2(C), (D)). For male (or female) inpatients with T1D aged 40–60 years, the PDN has 594 (or 544) nodes and 918 (or 953) links. There were more comorbid nodes and links in the 40–60 PDNs than the 1–20 PDNs. Based on the proportion test, both 1–20 PDNs of male and female had significantly less comorbidities in neoplasm category (140–239) than the 40–60 PDNs. The 1–20 PDN of male also had significantly less comorbidities in diseases of the musculoskeletal system and connective tissue (710–739) than the 40–60 PDN of male. The 1–20 PDN of male had significantly more comorbidities in endocrine, nutritional and metabolic diseases, and immunity disorders (240–279) than the 40–60 PDN of male. The details of T1D comorbid diseases for males and females were listed in the Table S6.

### PPI network of T1D

We constructed the PPI network under the STRING setting with 0.93 confidence based on active interaction sources from high-throughput lab experiments and previous knowledge in curated databases. The PPI network was generated from 48 genes related to T1D through the STRING database, covering 278 nodes and 919 edges (Figure S3). The number of edges is significantly larger than the expected for random network of the same size (*p*-value ≤ 10^−16^). It contained one giant component and several small separate components derived from seed proteins. The giant network consisted of 230 nodes and 865 links. The characteristics of the giant network, number of nodes (N), average degree (<*k*>), diameter (D), mean shortest path length (mspl), and average clustering coefficient (acc) were listed in Table S7. Furthermore, nodes with top 20% highest BC, DC, and CC were selected and listed in Table 2, Tables S8 and S9, respectively.

**Table 2:** The list of nodes with top 20% BC and their DC and CC values.

Rank	Protein	Degree	BC	CC
1	**CASP3**	45	**0.219802**	0.372358
2	**TGFB1**	21	**0.105624**	0.286967
3	CTNNB1	15	**0.095291**	0.379139
4	CD4	9	**0.087143**	0.298956
5	SRC	28	**0.074667**	0.391453
6	CASP8	21	**0.072508**	0.340774
7	UBC	40	**0.071289**	0.382304
8	EGFR	22	**0.067592**	0.373573
9	**AGT**	11	**0.066604**	0.250273
10	APP	7	**0.052618**	0.311141
11	SHC1	23	**0.051452**	0.370550
12	TGFBR2	8	**0.051187**	0.329496
13	**IGF1R**	22	**0.050850**	0.348554
14	CBL	27	**0.048630**	0.369355
15	PIK3R1	25	**0.047302**	0.377265
16	LCK	20	**0.045433**	0.353395
17	SNCA	6	**0.044806**	0.337261
18	AGTR1	5	**0.041871**	0.286250
19	CREBBP	14	**0.041003**	0.333333
20	PTPN11	19	**0.036798**	0.360063
21	**TNF**	29	**0.036016**	0.344361
22	FYN	22	**0.035117**	0.358372
23	CASP1	2	**0.034475**	0.256726
24	CDC5L	12	**0.034223**	0.298177
25	TGFBR1	8	**0.033620**	0.322535
26	**CCR5**	10	**0.033599**	0.253039
27	**CBLB**	26	**0.033107**	0.355590
28	RELA	15	**0.032008**	0.330925
29	STAT3	16	**0.030426**	0.340267
30	UBB	30	**0.028880**	0.360063
31	**NOS2**	5	**0.026136**	0.296632
32	**CTLA4**	7	**0.026063**	0.287688
33	**IL18**	4	**0.026048**	0.205566
34	**TOR1A**	4	**0.026048**	0.254162
35	SYK	16	**0.025902**	0.333819
36	ESR1	9	**0.025628**	0.337261
37	BIRC2	22	**0.024823**	0.346970
38	MAPK14	9	**0.024158**	0.350153
39	CAV1	11	**0.023528**	0.342814
40	TRAF2	28	**0.023094**	0.342302
41	ZAP70	12	**0.022875**	0.340267
42	ARRB1	6	**0.021873**	0.303311
43	UBE2I	8	**0.021765**	0.327611
44	NFKBIA	13	**0.020635**	0.360630
45	IL16	2	**0.020558**	0.290609
46	PRKCD	12	**0.020103**	0.353941
Average		15.78261	0.045287	0.329266

The bold proteins were also seed proteins. There were 11 seed proteins.

**Figure 2:**
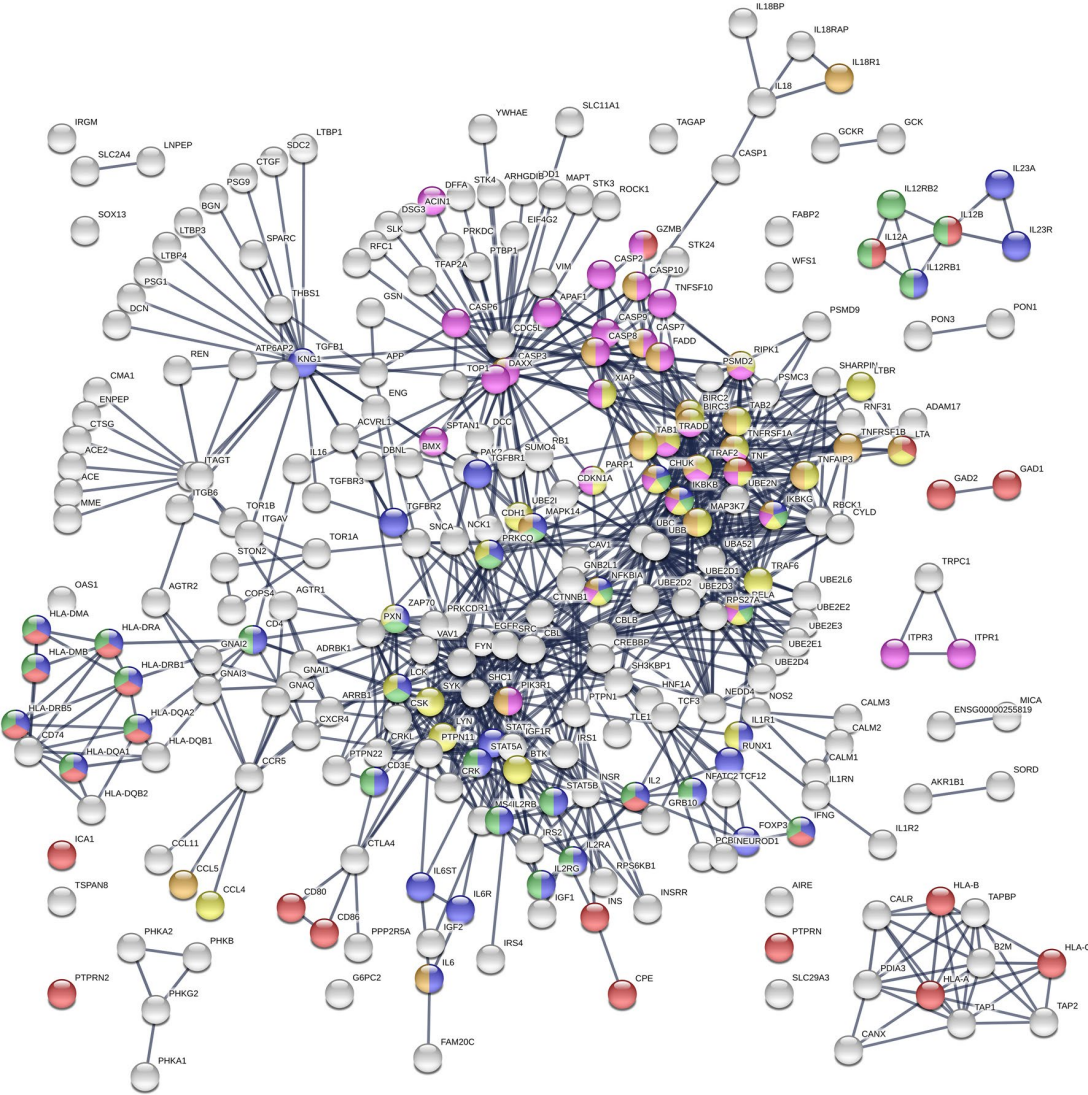
The KEGG pathways in the PPI network of T1D. The nodes involved in Th17 cell differentiation (hsa04659), type 1 diabetes mellitus (hsa04940), Th1 and Th2 cell differentiation (hsa04658), NF-kappa B signaling pathway (hsa04064), apoptosis (hsa04210), and TNF signaling pathway (hsa04668) were colored in blue, red, green, yellow, pink, and orange, respectively. The KEGG information were from KEGG database^22,23^. The PPI network was generated using STRING v11.0^26^ (https://string-db.org/).

Several KEGG pathways^33^ are involved in T1D. The nodes of the PPI network demonstrated 39 nodes for the pathway of Th17 cell differentiation, 26 nodes for type 1 diabetes mellitus, 30 nodes for Th1 and Th2 cell differentiation, 30 nodes for NF-kappa B signaling pathway, 31 nodes for Apoptosis, and 28 nodes for TNF signaling pathway (Table S10). The nodes of the PPI network involved in these KEGG pathways were marked in different colors (Fig. 2) and listed in Table S11.

### Backbone in the PPI network

In the PPI network, nodes with a BC value of the top 10% (BC ≥ 0.0344) were viewed as bottleneck nodes; nodes with a DC value of the top 10%t (DC ≥ 12) were considered as hubs. According to the result of the topological analysis, CASP3 was a hub (with the largest degree *k* = 45) and a bottleneck (with the highest BC = 0.219802) in the giant component. CASP3 also had the 6th highest CC value (CC = 0.372358), indicating that CASP3 was located at the center of the giant component. Because proteins with high BC are the heavily used intersections^13^, the top 20% highest BC nodes were then selected as key proteins (Table 2) to constitute the backbone of the giant component. The nodes in the backbone had much control over the nodes in the giant component and were extensively connected with their neighbors. The backbone network consisted of 46 nodes and 147 links (Figure S4). Among them, 13 proteins, CASP3, TGFB1, SRC, CASP8, UBC, EGFR, SHC1, IGF1R, CBL, PIK3R1, LCK, TNF, and FYN were date-hubs (hub-bottlenecks) which preferentially connect functional modules to each other^34^, whereas CTNNB1, CD4, AGT, APP, TGFBR2, SNCA, AGTR1, CREBBP, PTPN11, and CASP1 were bottlenecks but not hubs. In addition, UBC, UBB, TRAF2, CBLB, UBA52, IKBKG, RPS27A, BIRC2, MAP3K7, and BIRC3 were party-hubs (hub-nonbottlenecks) which preferentially act inside functional modules^34^. All of them worth further investigating the signaling pathways involved in T1D development. The nodes of the backbone network involved in the KEGG pathways were listed in the Table 3.

**Table 3:** The proteins of the backbone involved in the KEGG pathways.

Pathway	Description	#	Proteins involved in the KEGG pathway
hsa04659	Th17 cell differentiation	10	CD4, LCK, MAPK14, NFKBIA, RELA, STAT3, TGFB1, TGFBR1, TGFBR2, ZAP70 (blue)
hsa04940	Type I diabetes mellitus	1	TNF
hsa04658	Th1 and Th2 cell differentiation	6	CD4, LCK, MAPK14, NFKBIA, RELA, ZAP70 (green)
hsa04064	NF-kappa B signaling pathway	9	BIRC2, LCK, NFKBIA, RELA, SYK, TNF, TRAF 2, UBE2I, ZAP70 (yellow)
hsa04210	Apoptosis	8	BIRC2, **CASP3**, CASP8, NFKBIA, PIK3R1, RELA, TNF, TRAF2 (pink)
hsa04668	TNF signaling pathway	9	BIRC2, **CASP3**, CASP8, MAPK14, NFKBIA, PIK3R1, RELA, TNF, TRAF2 (orange)
hsa04060	Cytokine-cytokine receptor interaction	7	CCR5, EGFR, IL18, TGFB1, TGFBR1, TGFBR2, TNF
hsa04660	T cell receptor signaling pathway	11	CBLB, CD4, CTLA4, FYN, LCK, MAPK14, NFKBIA, PIK3R1, RELA, TNF, ZAP70
hsa04620	Toll-like receptor signaling pathway	6	CASP8, MAPK14, NFKBIA, PIK3R1, RELA, TNF
hsa04010	MAPK signaling pathway	11	ARRB1, **CASP3**, EGFR, IGF1R, MAPK14, RELA, TGFB1, TGFBR1, TGFBR2, TNF, TRAF2
hsa04910	Insulin signaling pathway	4	CBL, CBLB, PIK3R1, SHC1
hsa04630	Jak-STAT signaling pathway	5	CREBBP, EGFR, PIK3R1, PTPN11, STAT3
hsa04151	PI3K-Akt signaling pathway	5	EGFR, IGF1R, PIK3R1, RELA, SYK

### MiRNAs related to T1D

We searched the HMDD v3.2 database^32^ with disease “Diabetes Mellitus, Type 1” for miRNA related to T1D, and 32 entries with 25 miRNAs were collected (Table S12). Five miRNAs (miR-149-5p, miR-192, miR-21-5p, miR-23a-3p, and miR-23b-3p) were associated with T1D with causality. The miR-149, miR-23a, and miR-23b, downregulated by cytokines, regulated the expression of the proapoptotic Bcl-2 family members resulting in pancreatic β-cell destruction^35^. The miR-21 increased β-cell apoptosis in rats and humans through mRNA *BCL2* transcript degradation and inhibition of *BCL2* translation^30^. The miR-192 regulated β-cell development and inhibits insulin secretion via suppressing GLP-1 expression^36^.

The other miRNAs may play a crucial role in T1D pathogenesis. For example, serum let-7g expression reflected the decline of residual β-cell function and autoimmunity in T1D patients^37^. Eleven miRNAs (miR-100, miR-1275, miR-146a, miR-148a, miR-150, miR-181a, miR-21, miR-210, miR-24, miR-342, and miR-375) seem to be dysregulated in T1D patients^30^. Some miRNAs, miR-103a, miR-155, miR-200a, and miR-210 were confirmed as being upregulated in T1D patients; whilst miR-146a was downregulated in T1D patients^31^. Both miR-20a and miR-326 were upregulated in T1D patients^38^.

**Figure 3:**
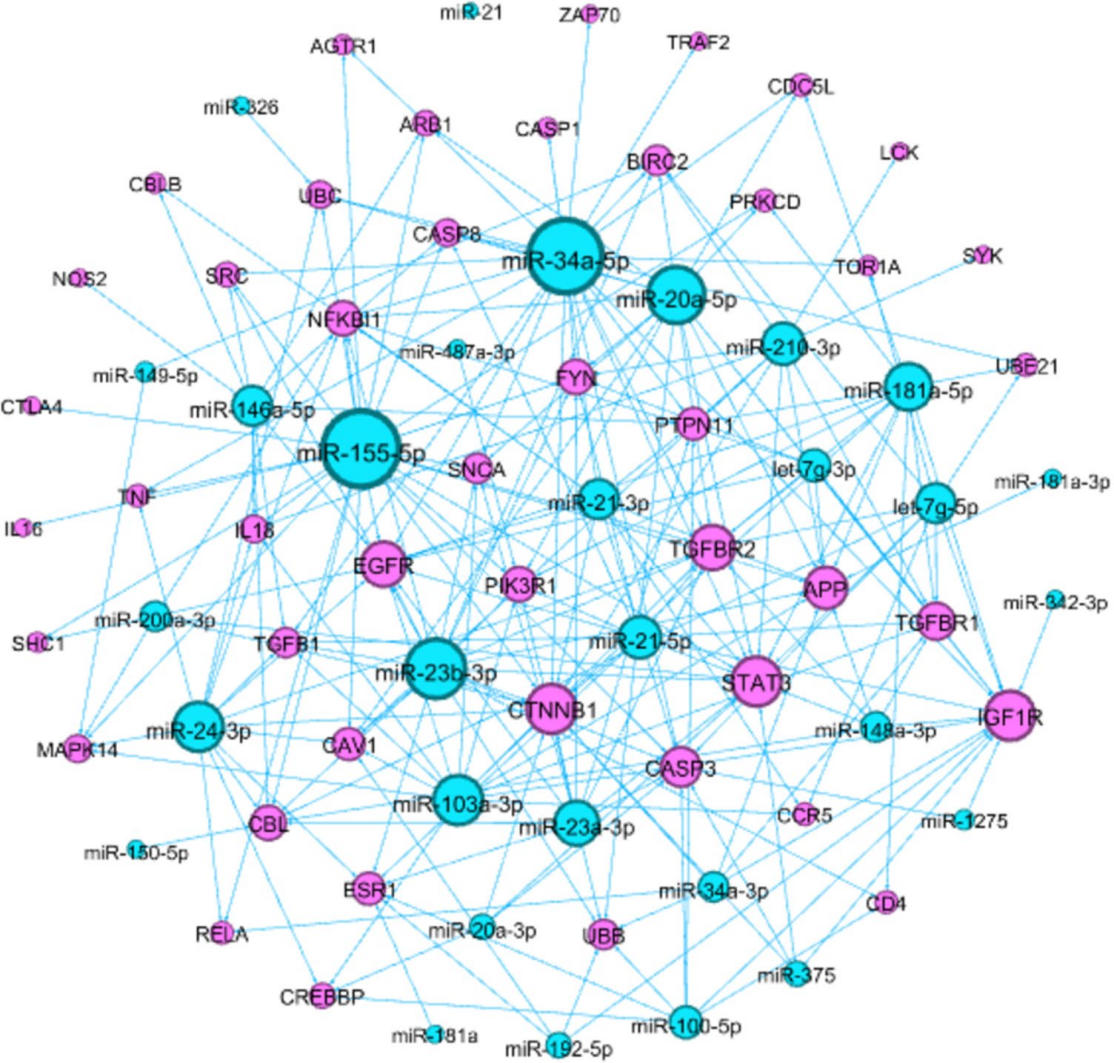
The interaction network between T1D-related miRNAs (blue) and backbone-proteins (red). The interaction network was generated using Gephi v0.9.2^29^ (https://gephi.org/).

Moreover, we searched the miRNet 2.0^39^, a miRNA-centric network visual analytics platform, for the miRNAs which target the backbone proteins. We took the intersection of this miRNet-miRNA set and the HMDD-T1D-miRNA set to form a list of the T1D-related miRNAs targeting backbone proteins (Table 4). Genes APP, CASP3, CTNNB1, EGFR, IGF1R, STAT3, and TGFBR2 were hub proteins which were targeted by more than 10 T1D-related miRNAs, whereas miRNAs miR-155-5p, miR-34a-5p, miR-23b-3p, miR-20a-5p, miR-103a-3p, miR-24-3p, miR-181a-5p, and miR-23a-3p were hub miRNAs which targeted more than 12 backbone proteins in the gene-miRNA network (Fig. 3).

**Table 4:** The T1D-related miRNAs targeting backbone proteins.

Proteins	#	miRNAs
AGT	0	
AGTR1	2	miR-155-5p, miR-34a-5p
APP	**11**	let-7g-3p, miR-103a-3p, miR-148a-3p, miR-155-5p, miR-181a-3p, miR-181a-5p, miR-20a-5p, miR-210-3p, miR-21-3p, miR-23b-3p, miR-34a-3p
ARRB1	4	miR-146a-5p, miR-155-5p, miR-20a-5p, miR-34a-5p
BIRC2	6	let-7g-5p, miR-149-5p, miR-20a-5p, miR-210-3p, miR-23b-3p, miR-34a-5p
CASP1	2	miR-21-3p, miR-34a-5p
CASP3	**10**	let-7g-5p, miR-100-5p, miR-1275, miR-155-5p, miR-20a-3p, miR-21-3p, miR-23a-3p, miR-23b-3p, miR-34a-5p, miR-375
CASP8	5	miR-146a-5p, miR-155-5p, miR-20a-5p, miR-21-5p, miR-34a-5p
CAV1	7	miR-103a-3p, miR-155-5p, miR-192-5p, miR-20a-5p, miR-210-3p, miR-23b-3p, miR-24-3p
CBL	8	let-7g-5p, miR-146a-5p, miR-148a-3p, miR-150-5p, miR-155-5p, miR-23a-3p, miR-23b-3p, miR-24-3p
CBLB	2	miR-146a-5p, miR-21-3p
CCR5	3	let-7g-5p, miR-103a-3p, miR-21-3p
CD4	3	miR-100-5p, miR-181a-5p, miR-23b-3p
CDC5L	3	miR-181a-5p, miR-20a-5p, miR-34a-5p
CREBBP	4	miR-100-5p, miR-103a-3p, miR-20a-3p, miR-24-3p
CTLA4	1	miR-155-5p
CTNNB1	**14**	miR-103a-3p, miR-155-5p, miR-181a-5p, miR-200a-3p, miR-20a-3p, miR-210-3p, miR-21-3p, miR-21-5p, miR-23a-3p, miR-23b-3p, miR-24-3p, miR-34a-3p, miR-34a-5p, miR-375
EGFR	**12**	let-7g-3p, miR-103a-3p, miR-146a-5p, miR-155-5p, miR-181a-5p, miR-200a-3p, miR-21-3p, miR-21-5p, miR-23a-3p, miR-23b-3p, miR-24-3p, miR-34a-5p
ESR1	7	miR-100-5p, mir-181a, miR-192-5p, miR-21-5p, miR-23a-3p, miR-23b-3p, miR-24-3p
FYN	8	let-7g-3p, miR-155-5p, miR-20a-5p, miR-210-3p, miR-21-5p, miR-23a-3p, miR-23b-3p, miR-34a-5p
IGF1R	**14**	let-7g-3p, let-7g-5p, miR-100-5p, miR-103a-3p, miR-1275, miR-148a-3p, miR-181a-5p, miR-192-5p, miR-20a-5p, miR-21-5p, miR-342-3p, miR-34a-3p, miR-34a-5p, miR-375
IL16	1	miR-155-5p
IL18	5	miR-103a-3p, miR-146a-5p, miR-155-5p, miR-210-3p, miR-24-3p
LCK	1	miR-210-3p
MAPK14	5	miR-103a-3p, miR-149-5p, miR-155-5p, miR-200a-3p, miR-24-3p
NFKBIA	8	leg-7g-3p, miR-155-5p, miR-200a-3p, miR-20a-5p, miR-21-3p, miR-23b-3p, miR-24-3p, miR-34a-5p
NOS2	1	miR-146a-5p
PIK3R1	8	miR-103a-3p, miR-155-5p, miR-181a-5p, miR-20a-5p, miR-21-5p, miR-23a-3p, miR-23b-3p, miR-487a-3p
PRKCD	3	miR-155-5p, miR-181a-5p, miR-20a-5p
PTPN11	7	miR-100-5p, miR-146a-5p, miR-181a-5p, miR-210-3p, miR-21-3p, miR-23a-3p, miR-34a-5p
RELA	3	miR-155-5p, miR-24-3p, miR-34a-4p
SHC1	2	miR-155-5p, miR-200a-3p
SNCA	6	miR-103a-3p, miR-155-5p, miR-20a-5p, miR-23a-3p, miR-23b-3p, miR-34a-5p
SRC	4	miR-146a-5p, miR-155-5p, miR-23b-3p, miR-34a-5p
STAT3	**14**	let-7g-5p, miR-148a-3p, miR-155-5p, miR-181a-5p, miR-200a-3p, miR-20a-3p, miR-20a-5p, miR-21-3p, miR-21-5p, miR-210-3p, miR-23a-3p, miR-23b-3p, miR-34a-5p, miR-375
SYK	1	miR-210-3p
TGFB1	6	miR-103a-3p, miR-146a-5p, miR-21-5p, miR-23b-3p, miR-24-3p, miR-34a-5p
TGFBR1	**9**	let-7g-3p, let-7g-5p, miR-103a-3p, miR-148a-3p, miR-181a-5p, miR-20a-5p, miR-210-3p, miR-21-5p, miR-34a-3p
TGFBR2	**12**	let-7g-3p, let-7g-5p, miR-103a-3p, miR-148a-3p, miR-155-5p, miR-181a-5p, miR-20a-5p, miR-21-5p, miR-23a-3p, miR-23b-3p, miR-24-3p, miR-34a-5p
TNF	3	miR-155-5p, miR-24-3p, miR-34a-5p
TOR1A	2	let-7g-5p, miR-34a-5p
TRAF2	1	miR-34a-5p
UBB	6	miR-100-5p, miR-192-5p, miR-20a-5p, miR-23a-3p, miR-23b-3p, miR-34a-3p
UBC	5	miR-155-5p, miR-20a-5p, miR-24-3p, miR-326, miR-34a-5p
UBE2I	3	let-7g-5p, miR-181a-5p, miR-34a-5p
ZAP70	1	miR-34a-5p

## Discussion

To address a global view of T1D, we analyzed medical claims of Taiwan NHIRD, gene data of the OMIM, protein interactions of the STRING, pathway information of the KEGG, and miRNA data of the HMDD. We developed a data repository linking medical claims and interactome studies in T1D. The main limitation of our study was not knowing where the T1D inpatients were in their disease course, e.g. at initial diagnosis or the number of years having suffered from T1D. Studies like TEDDY^40^ are designed to study disease initiation and progression to the development of clinical T1D. The inferred results of our work using medical claims can be regarded as relevant but not causal. By multifaceted information including genes, proteins, miRNAs, pathways, and medical claims, we elucidated multidimensionality of T1D heterogeneity and provided some hints for further research.

Both the PDNs of T1D males and females had more nodes in neoplasms (140–239), endocrine, nutritional and metabolic diseases, and immunity disorders (240–279), diseases of the nervous system and the sense organs (320–389), diseases of the circulatory system (390–459), and disease of the digestive system (520–579). Additionally, female inpatients have more comorbidities in the diseases of the genitourinary system (580–629). Furthermore, anemia caused by chronic renal disease, hypertensive renal disease, peripheral arterial disease with ulceration, nephritis and nephropathy, and chronic renal failure were correlated to T1D in men and women. Women with T1D aged 1–20 years old had comorbidity of peripheral angiopathy, whereas men with T1D aged 1–20 years old had comorbidities of cholesteatoma, orthostatic hypotension, decubitus ulcer, and osteomyelitis. Comorbid diseases of T1D such as anemia, atherosclerosis in peripheral vessels, cellulitis and abscess, decubitus ulcer, osteomyelitis, renal diseases, ureteral diseases, and vitreous diseases were reported in previous researches^1,10,11^. Nevertheless, cholesteatoma, orthostatic hypotension, and nonunion of fracture were novel comorbid diseases of T1D.

In our PPI network (Figures S1, S2), CASP3 is a hub and bottleneck protein. CASP3, one downstream effector caspase, and CASP8, one upstream initiator caspase, are members of caspases family and are both in the T1D backbone network. Caspases as cysteine-aspartyl specific proteases play key roles in β-cell apoptosis which is a fundamental process involved in the pathogenesis of T1D^41^. CASP3-mediated β-cell apoptosis is a necessary condition for T-cell priming which is a key initiating event to T1D^42^. CASP8 is critical for β-cell apoptosis in T1D and T2D and in maintaining β-cell mass and insulin secretion under physiological conditions^43^.

Transforming growth factor beta (TGF-β) is one of the factors involved in the cellular growth, differentiation and migration, and apoptosis^44^. TGFB1 could be a response to immuno‐inflammatory activation present at the onset of T1D^45^. It displays potent strong immunomodulatory activity and is involved in the control of autoimmune diseases, therefore it may aid in development of therapeutics to prevent the onset of autoimmunity, including T1D^44^.

CD4 belongs to the family of CD (cluster of differentiation) antigens which are cell surface-expressed antigens defined by monoclonal antibodies providing targets for immunophenotyping of cells^46^. It has an impact on immune function and carcinogenesis and is called T helper cell. The antigen is associated with a number of autoimmune diseases such as vitiligo and T1D^46^. In an animal study, TGF-β1 derived from T cells acts on diabetogenic CD4 + T cells, but not Foxp3 + Treg cells, to control Th1 cell differentiation and spontaneous T1D development^47^.

TNF is the cytokine which is mainly secreted by macrophages. Tumor necrosis factor-alpha (TNF-α) as a pro-inflammatory cytokine, participates in the regulation of several biological processes including cell apoptosis. It is suspected to relate to T1D pathogenesis^48^. Serum TNF-α level in T1D patients has significantly elevated among all age, disease duration and ethnicity groups. In addition, TNFRSF1A, a ubiquitous membrane receptor binding TNF-α, is associated with chronic renal failure^49^, which had comorbidity correlation with T1D in the PDNs. Circulating level of TNFRSF1A is considered as a biomarker of glomerular filtration rate decline in T1D patients.

Recently, studies on the renin angiotensin system (RAS) have shed light on the contribution of the RAS to the complications of T1D. The RAS includes circulating renin, acting on angiotensinogen (AGT) to produce angiotensin I (Ang I) and peptide angiotensin II (Ang II)^50^. An increase in the expression of AGT mRNA and in the Ang II synthesis may contribute to the glomerular sclerosis observed in diabetic nephropathy^51^. The IGF1R is generally considered as a growth factor receptor, and has important metabolic effects in many organisms. Once activated, the IGF1R will lead to glucose and lipid metabolism^52^. The IGF1R is involved in several signaling pathways (MAPK signaling pathway, PI3-kinase/PKB pathway, etc.), therefore it is related to T1D.

In the PPI network, six pathways related to T1D pathogenesis were marked in different colors. Three of them were also related to chronic renal failure, which had comorbidity correlations with T1D in the PDNs, including Th17 cell differentiation^53^, NF-kappa B signaling pathway^54^, and TNF signaling pathway^55^. Moreover, plenty of diseases pathways, such as pathways in cancer, toxoplasmosis, HTLV-1 infection, herpes simplex infection, Kaposi’s sarcoma-associated herpesvirus infection, Chagas disease (American trypanosomiasis), inflammatory bowel disease (IBD), Epstein-Barr virus infection, tuberculosis, allograft rejection, measles, Leishmaniasis, viral myocarditis, hepatitis B, influenza A, chronic myeloid leukemia, and autoimmune thyroid disease were involved in T1D PPI network (Table S10). Most of them have been confirmed by previous studies to be related to T1D^56–59^.

Several recent studies have identified some key genes and pathways for T1D using documented genes in literature^60^, selecting differentially expressed genes from microarray data^4,61^, combining GWAS statistics with gene expression profiles^62^, or mining RNA-seq datasets^63^. All these studies indicated that the genes and pathways involved in the immune system were key to the progression of T1D, which was consistent with our findings. Immune-linked biological pathways such as apoptosis, cytokine-cytokine receptor interaction, regulation of immune response, etc. were commonly highlighted, although the identified genes were somewhat different for different research purposes. Some solely focused on T1D-assocated genes and pathways^60,62^, another aimed at the interaction between peripheral blood mononuclear cells and pancreatic β-cells^61^, and the others were interested in T1D, multiple sclerosis, and other autoimmune diseases^4,63^. Compared to them, we investigated the genes and pathways related to T1D as well as chronic renal failure that had comorbidity correlations with T1D in our PDNs.

The biomarkers of T1D often use autoantibodies against islet antigen, such as GADA (glutamate decarboxylase), IAA (insulin), ICA (islet cells), IA-2, IA-2β (tyrosine phosphatase), and ZnT8 (zinc transporter 8). Nevertheless, because autoantibodies appear relatively late and there are many false positives, additional biomarkers for T1D are needed^19^. Recently, miRNAs in serum, plasma or blood cells have been developed to predict the development and progression of T1D. Many of miRNAs targeted mRNAs (miRNA-mRNA interactions) are associated with diabetes pathogenesis. The miR-181a-5p targets mRNAs for CD4^+^ and CD8^+^ in the T cell receptor (TCR) signaling pathway (hsa04660)^30^. The miR-181a-5p and miR-21-5p bind to *STAT3* mRNA in the Jak-STAT signaling pathway (hsa04630)^30^. MicroRNAs miR-23a-3p, miR-23b-3p, and miR-149-5p regulate proapoptotic Bcl-2 proteins DP5 and PUMA and consequent human β-cell apoptosis^35^. More studies focused on clarifying the specific role of miRNAs in pancreatic islets and islet-infiltrating immune cells^31,36–38^. These pioneering studies demonstrate the potential of miRNAs as biomarkers for T1D, but the heterogeneity of the results need to be verified by more studies.

## Conclusion

Advances in science and technology have enabled the study of biological systems in their integrity. By understanding the multifaceted complexity of biological systems, predicting the risk of disease development and controlling disease progression to prevent complications will be realized. We used the medical records of Taiwan NHRID and online databases of genes, proteins, and miRNAs to have a systemic view of T1D. There were common comorbid diseases such as anemia, hypertension, vitreous diseases, renal diseases, and atherosclerosis; whereas there were novel comorbid diseases such as cholesteatoma, orthostatic hypotension, and nonunion of fracture in the PDN. We constructed the PPI network derived from T1D related genes. In the PPI network, CASP3 is a date-hub protein involved in apoptosis, TNF signaling, and MAPK pathways. The protein TNF is also a date-hub protein involving T1DM, NFKB, apoptosis, cytokine-cytokine, TCR, TLR, TNF signaling, and MAPK pathways. Moreover, we connected T1D related miRNAs with backbone proteins to understand the relationships between miRNAs and backbone proteins. CTNNB1, IGF1R, and STAT3 were hub proteins, whereas miR-155-5p, miR-34a-5p, miR-23-3p, and miR-20a-5p were hub miRNAs in the gene-miRNA network. Multiple levels of information including genetic, protein, and clinical data resulted in multiple results, which suggests the complementarity of multiple sources. With the integration of multifaceted information, it will shed light on the mechanisms underlying T1D; the provided data and repository has utility in understanding phenotypic disease networks for the potential development of comorbidities in T1D patients as well as the clues for further research on T1D comorbidities.

## Supplementary Information

Supplementary Information.
