# Supplementary material for: Elucidate multidimensionality of type 1 diabetes mellitus heterogeneity by multifaceted information
Source: Sci Rep. 2021 Oct 25;11:20965. doi: 10.1038/s41598-021-00388-2 (PMC8545927; doi:10.1038/s41598-021-00388-2)
Supplement: Supplementary file 1 — Supplementary Information. [file 41598_2021_388_MOESM1_ESM.pdf]

Supplementary Information

Elucidate Multidimensionality of Type 1 Diabetes Mellitus Heterogeneity by Multifaceted Information

Shaw-Ji Chen[1,2], Jen-Liang Cheng[3], Sheng-An Lee[4], Tse-Yi Wang[3], Jyy-Yu Jang[3], Kuang-Chi Chen[3*]

[1] Department of Psychiatry, Taitung, Mackay Memorial Hospital, Taiwan

[2] Department of Medicine, Mackay Medical College, New Taipei City, Taiwan

[3] Department of Medical Informatics, Tzu Chi University, Hualien, Taiwan

[4] Department of Health Industry Management, Kainan University, Taoyuan, Taiwan

[*] To whom correspondence should be addressed: chichen6@mail.tcu.edu.tw

Legend of Tables

Table S1. The disease categories of ICD-9-CM in the PDNs.

Table S2. The T1D related genes from the OMIM.

Table S3. The GO biological processes of the T1D related genes.

Table S4. The number of nodes for each disease category in the PDNs of T1D and control.

Table S5. The number of nodes for each disease category in the PDNs of 1-20 T1D and 40-60 T1D.

Table S6. The comorbid diseases associated with T1D.

Table S7. The characteristics of the giant component and backbone network.

Table S8. The list of nodes with top 20% DC and their BC & CC values.

Table S9. The list of nodes with top 20% CC and their DC & BC values.

Table S10. The top 50 KEGG pathways of enrichment in the PPI network of T1D.

Table S11. The proteins of the PPI network involved in the KEGG pathways.

Table S12. The miRNAs associated with T1D.

Legend of Figures

Figure S1. The PDNs of control group for (A) Male and (B) Female inpatients.

Figure S2. The PDNs of T1D for (A) 1-20 male, (B) 1-20 female, (C) 40-60 male, and (D) 40-60 female inpatients.

Figure S3. The PPI network of T1D.

Figure S4. The backbone network.

Table S1. The disease categories of ICD-9-CM in the PDNs.

Chapter	ICD-9-CM	Title
**I**	001-139	Infectious and Parasitic Diseases
**II**	140-239	Neoplasms
**III**	240-279	Endocrine, Nutritional and Metabolic Diseases, and Immunity Disorders
**IV**	280-289	Diseases of the Blood and Blood-forming Organs
**V**	290-319	Mental Disorders
**VI**	320-389	Diseases of the Nervous System and Sense Organs
**VII**	390-459	Diseases of the Circulatory System
**VIII**	460-519	Diseases of the Respiratory System
**IX**	520-579	Diseases of the Digestive System
**X**	580-629	Diseases of the Genitourinary System
**XII**	680-709	Diseases of the Skin and Subcutaneous Tissue
**XIII**	710-739	Diseases of the Musculoskeletal System and Connective Tissue
**XIV**	740-759	Congenital Anomalies
**Excluded disease chapters**		
**XI**	630-679	Complications of Pregnancy, Childbirth, and the Puerperium
**XV**	760-779	Certain Conditions originating in the Perinatal Period
**XVI**	780-799	Symptoms, Signs and Ill-defined Conditions
**XVII**	800-999	Injury and Poisoning
	E800-E999	Supplementary Classification of External Causes of Injury and Poisoning
	V01-V82	Supplementary Classification of Factors influencing Health Status and Contact with Health Services

Table S2. The T1D related genes from the OMIM.

#	Protein	Name
1	ACE	ANGIOTENSIN I-CONVERTING ENZYME
2	AGT	ANGIOTENSINOGEN
3	AIRE	AUTOIMMUNE REGULATOR
4	AKR1B1	ALDO-KETO REDUCTASE FAMILY 1, MEMBER B1
5	CASP3	CASPASE 3, APOPTOSIS-RELATED CYSTEINE PROTEASE
6	CBLB	CAS-BR-M MURINE ECOTROPIC RETROVIRAL TRANSFORMING SEQUENCE B
7	CCR5	CHEMOKINE, CC MOTIF, RECEPTOR 5
8	CTLA4	CYTOTOXIC T LYMPHOCYTE-ASSOCIATED 4
9	FABP2	FATTY ACID-BINDING PROTEIN 2
10	FOXP3	FORKHEAD BOX P3
11	G6PC2	GLUCOSE-6-PHOSPHATASE, CATALYTIC, 2
12	GAD2	GLUTAMATE DECARBOXYLASE 2
13	GCK	GLUCOKINASE;
14	HLA-DQA1	MAJOR HISTOCOMPATIBILITY COMPLEX, CLASS II, DQ ALPHA-1
15	HLA-DQB1	MAJOR HISTOCOMPATIBILITY COMPLEX, CLASS II, DQ BETA-1
16	HLA-DRA	MAJOR HISTOCOMPATIBILITY COMPLEX, CLASS II, DR ALPHA
17	HNF1A	HNF1 HOMEOBOX A
18	ICA1	ISLET CELL AUTOANTIGEN 1
19	IGF1R	INSULIN-LIKE GROWTH FACTOR I RECEPTOR
20	IL12B	INTERLEUKIN 12B
21	IL18	INTERLEUKIN 18
22	IL1RN	INTERLEUKIN 1 RECEPTOR ANTAGONIST
23	IL2RA	INTERLEUKIN 2 RECEPTOR, ALPHA
24	IL6	INTERLEUKIN 6
25	INS	INSULIN
26	IRGM	IMMUNITY-RELATED GTPase FAMILY, M
27	ITPR3	INOSITOL 1,4,5-TRISPHOSPHATE RECEPTOR, TYPE 3
28	MICA	MAJOR HISTOCOMPATIBILITY COMPLEX CLASS I CHAIN-RELATED GENE A
29	NEUROD1	NEUROGENIC DIFFERENTIATION 1
30	NOS2A	NITRIC OXIDE SYNTHASE 2A (STRING, GO: NOS2)
31	OAS1	2-PRIME,5-PRIME-OLIGOADENYLATE SYNTHETASE 1
32	PHKG2	PHOSPHORYLASE KINASE, TESTIS/LIVER, GAMMA-2
33	PON1	PARAOXONASE 1
34	PTPN22	PROTEIN TYROSINE PHOSPHATASE, NONRECEPTOR-TYPE, 22
35	PTPRN	PROTEIN-TYROSINE PHOSPHATASE, RECEPTOR-TYPE, N
36	PTPRN2	PROTEIN-TYROSINE PHOSPHATASE, RECEPTOR-TYPE, N, POLYPEPTIDE 2
37	SLC11A1	SOLUTE CARRIER FAMILY 11 (PROTON-COUPLED DIVALENT METAL ION TRANSPORTER), MEMBER 1
38	SLC2A4	SOLUTE CARRIER FAMILY 2 (FACILITATED GLUCOSE TRANSPORTER), MEMBER 4
39	SLC29A3	EQUILIBRATIVE NUCLEOSIDE TRANSPORTER 3
40	SOX13	SRY-BOX 13
41	SUMO4	SMALL UBIQUITIN-LIKE MODIFIER 4
42	TAGAP	T-CELL ACTIVATION GTPase-ACTIVATING PROTEIN
43	TAP1	TRANSPORTER, ATP-BINDING CASSETTE, MAJOR HISTOCOMPATIBILITY COMPLEX, 1
44	TGFB1	TRANSFORMING GROWTH FACTOR, BETA-1
45	TNF	TUMOR NECROSIS FACTOR
46	TOR1A	TORSIN 1A
47	TSPAN8	TETRASPANIN 8
48	WFS1	WOLFRAMIN ER TRANSMEMBRANE GLYCOPROTEIN

Table S3. The GO biological processes of the T1D related genes.

GO biological process complete	Homo sapie	upload(52)	(expected)	(over/under)	(fold Enrich	(FDR)
**carbohydrate homeostasis (GO:0033500)**	**198**	**13**	**0.49**	**+**	**26.33**	**2.78E-11**
regulation of hormone levels (GO:0010817)	534	17	1.33	+	12.77	4.17E-11
**glucose homeostasis (GO:0042593)**	**197**	**13**	**0.49**	**+**	**26.46**	**5.23E-11**
response to organic substance (GO:0010033)	3027	32	7.55	+	4.24	5.51E-11
cellular response to organic substance (GO:0071310)	2360	28	5.89	+	4.76	3.07E-10
homeostatic process (GO:0042592)	1681	24	4.19	+	5.72	7.72E-10
immune system process (GO:0002376)	2851	29	7.11	+	4.08	2.09E-09
chemical homeostasis (GO:0048878)	1128	20	2.81	+	7.11	2.19E-09
response to chemical (GO:0042221)	4415	35	11.01	+	3.18	2.41E-09
response to oxygen-containing compound (GO:190170	1605	23	4	+	5.75	2.41E-09
cellular response to chemical stimulus (GO:0070887)	2929	29	7.3	+	3.97	3.76E-09
regulation of hormone secretion (GO:0046883)	277	12	0.69	+	17.37	6.49E-09
immune response (GO:0006955)	1934	24	4.82	+	4.98	7.07E-09
cytokine-mediated signaling pathway (GO:0019221)	700	16	1.75	+	9.17	1.13E-08
response to cytokine (GO:0034097)	1126	19	2.81	+	6.77	1.26E-08
regulation of protein secretion (GO:0050708)	386	13	0.96	+	13.5	1.26E-08
response to stress (GO:0006950)	3656	31	9.12	+	3.4	1.43E-08
positive regulation of leukocyte cell-cell adhesion (GO	235	11	0.59	+	18.77	1.67E-08
regulation of leukocyte cell-cell adhesion (GO:190303	320	12	0.8	+	15.04	2.07E-08
regulation of peptide secretion (GO:0002791)	417	13	1.04	+	12.5	2.40E-08
cellular response to cytokine stimulus (GO:0071345)	1038	18	2.59	+	6.95	2.51E-08
regulation of cell-cell adhesion (GO:0022407)	431	13	1.07	+	12.09	3.11E-08
regulation of immune system process (GO:0002682)	1734	22	4.32	+	5.09	3.14E-08
regulation of biological quality (GO:0065008)	4102	32	10.23	+	3.13	3.34E-08
cellular response to oxygen-containing compound (GC	1076	18	2.68	+	6.71	3.77E-08
defense response (GO:0006952)	1434	20	3.58	+	5.59	5.33E-08
regulation of secretion by cell (GO:1903530)	683	15	1.7	+	8.81	5.36E-08
positive regulation of cell-cell adhesion (GO:0022409	279	11	0.7	+	15.81	6.28E-08
response to biotic stimulus (GO:0009607)	1462	20	3.65	+	5.49	6.97E-08
response to lipid (GO:0033993)	842	16	2.1	+	7.62	7.76E-08
positive regulation of T cell activation (GO:0050870)	216	10	0.54	+	18.56	9.76E-08
cytokine production (GO:0001816)	152	9	0.38	+	23.74	1.05E-07
cell surface receptor signaling pathway (GO:0007166	2523	25	6.29	+	3.97	1.05E-07
cellular response to biotic stimulus (GO:0071216)	221	10	0.55	+	18.14	1.10E-07
lymphocyte activation (GO:0046649)	395	12	0.99	+	12.18	1.11E-07
regulation of peptide hormone secretion (GO:0090276	222	10	0.55	+	18.06	1.12E-07
regulation of secretion (GO:0051046)	739	15	1.84	+	8.14	1.13E-07
adaptive immune response (GO:0002250)	616	14	1.54	+	9.11	1.14E-07
regulation of localization (GO:0032879)	2796	26	6.97	+	3.73	1.25E-07
**positive regulation of adaptive immune response ba**	**105**	**8**	**0.26**	**+**	**30.55**	**1.39E-07**
response to lipopolysaccharide (GO:0032496)	318	11	0.79	+	13.87	1.64E-07
**positive regulation of adaptive immune response (G**	**110**	**8**	**0.27**	**+**	**29.16**	**1.88E-07**
regulation of T cell proliferation (GO:0042129)	169	9	0.42	+	21.35	1.91E-07
T cell activation (GO:0042110)	245	10	0.61	+	16.37	2.23E-07
response to external biotic stimulus (GO:0043207)	1430	19	3.57	+	5.33	2.39E-07
regulation of T cell activation (GO:0050863)	334	11	0.83	+	13.21	2.41E-07
response to other organism (GO:0051707)	1428	19	3.56	+	5.34	2.44E-07
response to molecule of bacterial origin (GO:0002237	336	11	0.84	+	13.13	2.45E-07
regulation of protein transport (GO:0051223)	670	14	1.67	+	8.38	2.52E-07
positive regulation of immune system process (GO:00	1100	17	2.74	+	6.2	2.52E-07

Table S4. The number of nodes for each disease category in the PDNs of T1D and control.

**ICD-9**	**Name of disease category**	**T1D Male # of nodes (%)**	**T1D Female # of nodes (%)**	**Control Male # of nodes (%)**	**Control Female # of nodes (%)**
001-139	Infectious and parasitic diseases	19 (3.36)*	21 (3.64)	45 (6.23)*	41 (5.33)
140-239	Neoplasms	**61 (10.78)**	**70 (12.13)**	**80 (11.08)**	**82 (10.66)**
240-279	Endocrine, nutritional and metabolic diseases, and immunity disorders	**69 (12.19)****	**54 (9.36)**	50 (6.93)**	58 (7.54)
280-289	Diseases of the blood and blood-forming organs	18 (3.18)	20 (3.47)	26 (3.60)	29 (3.77)
290-319	Mental disorders	27 (4.77)	27 (4.68)	35 (4.85)	39 (5.07)
320-389	Diseases of the nervous system and the nervous system	**74 (13.07)**	**87 (15.08)**	**102 (14.13)**	**107 (13.91)**
390-459	Diseases of the circulatory system	**62 (10.95)**	**67 (11.61)**	**88 (12.19)**	**93 (12.09)**
460-519	Diseases of the respiratory system	27 (4.77)	25 (4.33)	51 (7.06)	52 (6.76)
520-579	Diseases of the digestive system	**62 (10.95)**	**57 (9.88)**	**98 (13.57)**	**95 (12.35)**
580-629	Diseases of the genitourinary system	30 (5.30)***	**63 (10.92)*****	55 (7.62)	**71 (9.23)**
680-709	Diseases of the skin and subcutaneous tissue	41 (7.24)***	32 (5.55)	22 (3.05)***	26 (3.38)
710-739	Diseases of the musculoskeletal system and connective tissue	42 (7.42)	28 (4.85)	36 (4.99)	40 (5.20)
740-759	Congenital anomalies	34 (6.01)	26 (4.51)	34 (4.71)	36 (4.68)
Total		566 (100.00)	577 (100.00)	722 (100.00)	769 (100.00)

Compare the proportion of each disease category in one PDN with the proportion of the same disease category in another PDN by two-sample proportion test.
*: p-value < 0.05; **: p-value <0 0.01; ***: p-value < 0.001.

Table S5. The number of nodes for each disease category in the PDNs of 1-20 T1D and 40-60 T1D.

ICD-9	Name of disease category	1-20 Male # of nodes (%)	40-60 Male # of nodes (%)	1-20 Female # of nodes (%)	40-60 Female # of nodes (%)
001-139	Infectious and parasitic diseases	20 (6.19)	21 (3.54)	27 (7.38)	27 (4.96)
140-239	Neoplasms	15 (4.64)**	**59 (9.93)****	18 (4.92)**	**52 (9.56)****
240-279	Endocrine, nutritional and metabolic diseases, and immunity disorders	**46 (14.24)***	**49 (8.25)***	**46 (12.57)**	**53 (9.74)**
280-289	Diseases of the blood and blood-forming organs	12 (3.72)	22 (3.70)	15 (4.10)	20 (3.68)
290-319	Mental disorders	18 (5.57)	26 (4.38)	22 (6.01)	31 (5.70)
320-389	Diseases of the nervous system and sense organs	**46 (14.24)**	**83 (13.97)**	**58 (15.85)**	**71 (13.05)**
390-459	Diseases of the circulatory system	**41 (12.69)**	**84 (14.14)**	**38 (10.38)**	**61 (11.21)**
460-519	Diseases of the respiratory system	19 (5.88)	43 (7.24)	27 (7.38)	27 (4.96)
520-579	Diseases of the digestive system	**38 (11.76)**	**69 (11.62)**	**40 (10.93)**	**52 (9.56)**
580-629	Diseases of the genitourinary system	25 (7.74)	41 (6.90)*	**32 (8.74)**	**65 (11.95)***
680-709	Diseases of the skin and subcutaneous tissue	19 (5.88)	32 (5.39)	15 (4.10)	30 (5.51)
710-739	Diseases of the musculoskeletal system and connective tissue	12 (3.72)*	43 (7.24)*	13 (3.55)	30 (5.51)
740-759	Congenital anomalies	12 (3.72)	22 (3.70)	15 (4.10)	25 (4.60)
Total		323	594	366	544

Compare the proportion of each disease category in one PDN with the proportion of the same disease category in another PDN by two-sample proportion test.
*: *p*-value < 0.05; **: *p*-value <0 0.01; ***: *p*-value < 0.001.

Table S6. The comorbid diseases associated with T1D.

Age	Male	Female
**1-20**	320-389<br>- Cholesteatoma	
	390-459<br>- Orthostatic hypotension	390-459<br>- Peripheral angiopathy
	580-629<br>- Nephritis and nephropathy<br>- Calculus of ureter<br>- Ureteric obstruction	580-629<br>- Nephritis and nephropathy
	680-709<br>- Cellulitis and abscess<br>- Decubitus ulcer	
	710-739<br>- Osteomyelitis<br>- Malunion and nonunion of fracture	
**40-60**	280-289<br>- Anemia caused by chronic renal disease	280-289<br>- Anemia caused by chronic renal disease
	390-459<br>- Hypertensive renal disease<br>- Peripheral arterial disease w/ ulceration	
	580-629<br>- Nephritis and nephropathy	
**All ages**	280-289<br>- Anemia caused by chronic renal disease	280-289<br>- Anemia caused by chronic renal disease
		320-379<br>- Vitreous diseases
	390-459<br>- Hypertensive renal disease<br>- Peripheral arterial disease w/ ulceration	390-459<br>- Hypertensive renal disease<br>- Peripheral arterial disease w/ ulceration
	580-629<br>- Nephritis and nephropathy<br>- Chronic renal failure	580-629<br>- Nephritis and nephropathy<br>- Chronic renal failure

Table S7. The characteristics of the giant component and backbone network.

Symbol	Characteristic	Giant component	Backbone network
**N**	Number of nodes	230	46
**<*k*>**	Average degree	7.522	15.783*
**D**	Diameter	9	7
**mspl**	Mean shortest path length	3.68	2.581
**acc**	Average clustering coefficient	0.528	0.335

Note: The nodes of backbone network were selected as top 20% BC nodes from giant network
*: The mean degree of backbone-nodes in the giant component.

Table S8. The list of nodes with top 20% DC and their BC & CC values.

Rank	Protein	Degree	BC	CC
1	**CASP3**	**45**	0.219802	0.372358
2	UBC	**40**	0.071289	0.382304
3	UBB	**30**	0.02888	0.360063
4	**TNF**	**29**	0.036016	0.344361
5	SRC	**28**	0.074667	0.391453
6	TRAF2	**28**	0.023094	0.342302
7	CBL	**27**	0.048630	0.369355
8	**CBLB**	**26**	0.033107	0.35559
9	UBA52	**25**	0.013327	0.331404
10	PIK3R1	**25**	0.047302	0.377265
11	IKBKG	**24**	0.004510	0.332849
12	SHC1	**23**	0.051452	0.370550
13	RPS27A	**23**	0.006300	0.32808
14	FYN	**22**	0.035117	0.358372
15	EGFR	**22**	0.067592	0.373573
16	BIRC2	**22**	0.024823	0.346970
17	**IGF1R**	**22**	0.050850	0.348554
18	TNFRSF1A	**22**	0.011403	0.324823
19	CASP8	**21**	0.072508	0.340774
20	**TGFB1**	**21**	0.105624	0.286967
21	MAP3K7	**20**	0.005641	0.337758
22	BIRC3	**20**	0.017142	0.342814
23	LCK	**20**	0.045433	0.353395
24	RIPK1	**19**	0.005842	0.316298
25	PTPN11	**19**	0.036798	0.360063
26	IKBKB	**17**	0.006170	0.334795
27	TNFAIP3	**17**	0.001979	0.298956
28	TRAF6	**16**	0.009486	0.312415
29	SYK	**16**	0.025902	0.333819
30	STAT3	**16**	0.030426	0.340267
31	CTNNB1	**15**	0.095291	0.379139
32	RELA	**15**	0.032008	0.330925
33	XIAP	**15**	0.019335	0.340267
34	CHUK	**15**	0.005459	0.328080
35	TAB2	**14**	0.009290	0.309042
36	UBE2D1	**14**	0.002597	0.332849
37	INSR	**14**	0.014742	0.317614
38	LYN	**14**	0.009201	0.343328
39	CREBBP	**14**	0.041003	0.333333
40	CASP7	**13**	0.004889	0.314560
41	RBCK1	**13**	0.001055	0.304117
42	UBE2D2	**13**	0.005936	0.338757
43	IRS1	**13**	0.004678	0.320728
44	NFKBIA	**13**	0.020635	0.360630
45	ZAP70	**12**	0.022875	0.340267
46	CDC5L	**12**	0.034223	0.298177
47	CRK	**12**	0.001379	0.316298
48	PRKCD	**12**	0.020103	0.353941
49	CYLD	**12**	0.000377	0.292092
50	CSK	**12**	0.010708	0.329023

Note: The bold proteins were also seed proteins. There were 5 seed proteins.

Table S9. The list of nodes with top 20% CC and their DC & BC values.

**Rank**	**Protein**	**Degree**	**BC**	**CC**
1	SRC	28	0.074667	**0.391453**
2	UBC	40	0.071289	**0.382304**
3	CTNNB1	15	0.095291	**0.379139**
4	PIK3R1	25	0.047302	**0.377265**
5	EGFR	22	0.067592	**0.373573**
6	**CASP3**	45	0.219802	**0.372358**
7	SHC1	23	0.051452	**0.370550**
8	CBL	27	0.048630	**0.369355**
9	NFKBIA	13	0.020635	**0.360630**
10	PTPN11	19	0.036798	**0.360063**
11	UBB	30	0.028880	**0.360063**
12	FYN	22	0.035117	**0.358372**
13	**CBLB**	26	0.033107	**0.355590**
14	PRKCD	12	0.020103	**0.353941**
15	LCK	20	0.045433	**0.353395**
16	MAPK14	9	0.024158	**0.350153**
17	**IGF1R**	22	0.050850	**0.348554**
18	BIRC2	22	0.024823	**0.346970**
19	CDH1	5	0.006924	**0.344880**
20	**TNF**	29	0.036016	**0.344361**
21	LYN	14	0.009201	**0.343328**
22	CAV1	11	0.017142	**0.342814**
23	BIRC3	20	0.023528	**0.342814**
24	TRAF2	28	0.023094	**0.342302**
25	PRKCQ	8	0.018373	**0.341282**
26	CASP8	21	0.072508	**0.340774**
27	STAT3	16	0.022875	**0.340267**
28	ZAP70	12	0.019335	**0.340267**
29	XIAP	15	0.030426	**0.340267**
30	UBE2D2	13	0.005936	**0.338757**
31	MAP3K7	20	0.005641	**0.337758**
32	SNCA	6	0.025628	**0.337261**
33	ESR1	9	0.044806	**0.337261**
34	GNB2L1	8	0.010312	**0.335286**
35	IKBKB	17	0.006170	**0.334795**
36	VAV1	10	0.005956	**0.334307**
37	SYK	16	0.025902	**0.333819**
38	CREBBP	14	0.041003	**0.333333**
39	PARP1	5	0.004510	**0.332849**
40	IKBKG	24	0.002597	**0.332849**
41	UBE2D1	14	0.008536	**0.332849**
42	UBA52	25	0.013327	**0.331404**
43	RELA	15	0.032008	**0.330925**
44	TGFBR2	8	0.051187	**0.329496**
45	CSK	12	0.005783	**0.329023**
46	PAK2	5	0.006728	**0.329023**

Note: The bold proteins were also seed proteins. There were 4 seed proteins.

Table S10. The top 50 KEGG pathways of enrichment in the PPI network of T1D.

#	Pathway	Description	Count in gene set	FDR
1	hsa04659	**Th17 cell differentiation**	39 of 102	2.15E-37
2	hsa05200	Pathways in cancer	64 of 515	5.82E-37
3	hsa05145	Toxoplasmosis	36 of 109	5.58E-33
4	hsa04940	**Type I diabetes mellitus (T1DM)**	26 of 40	1.13E-29
5	hsa05166	HTLV-I infection	43 of 250	1.26E-29
6	hsa05168	Herpes simplex infection	38 of 181	6.45E-29
7	hsa04658	**Th1 and Th2 cell differentiation**	30 of 88	5.30E-28
8	hsa04064	**NF-kappa B signaling pathway (NFKB)**	30 of 93	1.81E-27
9	hsa05167	Kaposi's sarcoma-associated herpesvirus infection	35 of 183	1.56E-25
10	hsa05142	Chagas disease (American trypanosomiasis)	29 of 101	2.04E-25
11	hsa05321	Inflammatory bowel disease (IBD)	25 of 62	6.38E-25
12	hsa05169	Epstein-Barr virus infection	35 of 194	6.66E-25
13	hsa04210	**Apoptosis**	31 of 135	8.63E-25
14	hsa04668	**TNF signaling pathway**	28 of 108	1.24E-23
15	hsa05152	Tuberculosis	32 of 172	3.46E-23
16	hsa04621	NOD-like receptor signaling pathway	30 of 166	1.99E-21
17	hsa04060	**Cytokine-cytokine receptor interaction**	34 of 263	3.75E-20
18	hsa05330	Allograft rejection	18 of 35	1.32E-19
19	hsa05162	Measles	26 of 133	2.22E-19
20	hsa04612	Antigen processing and presentation	21 of 66	2.22E-19
21	hsa04380	Osteoclast differentiation	25 of 124	5.85E-19
22	hsa04660	**T cell receptor signaling pathway (TCR)**	23 of 99	1.15E-18
23	hsa05332	Graft-versus-host disease	17 of 36	3.86E-18
24	hsa05140	Leishmaniasis	20 of 70	8.63E-18
25	hsa04062	Chemokine signaling pathway	27 of 181	1.49E-17
26	hsa04620	**Toll-like receptor signaling pathway (TLR)**	22 of 102	2.47E-17
27	hsa04010	**MAPK signaling pathway**	32 of 293	3.50E-17
28	hsa05416	Viral myocarditis	18 of 56	7.27E-17
29	hsa05205	Proteoglycans in cancer	27 of 195	7.27E-17
30	hsa05161	Hepatitis B	24 of 142	8.42E-17
31	hsa05203	Viral carcinogenesis	26 of 183	1.55E-16
32	hsa04068	FoxO signaling pathway	23 of 130	1.58E-16
33	hsa05164	Influenza A	25 of 168	2.32E-16
34	hsa05220	Chronic myeloid leukemia	19 of 76	3.82E-16
35	hsa04920	Adipocytokine signaling pathway	18 of 69	1.32E-15
36	hsa04622	RIG-I-like receptor signaling pathway	18 of 70	1.59E-15
37	hsa04910	**Insulin signaling pathway**	22 of 134	2.79E-15

38	hsa04657	IL-17 signaling pathway	19 of 92	7.24E-15
39	hsa04217	Necroptosis	22 of 155	3.98E-14
40	hsa05320	Autoimmune thyroid disease	15 of 49	5.32E-14
41	hsa04630	**Jak-STAT signaling pathway**	22 of 160	6.82E-14
42	hsa04931	Insulin resistance	19 of 107	7.40E-14
43	hsa05222	Small cell lung cancer	18 of 92	8.34E-14
44	hsa04614	Renin-angiotensin system	12 of 23	1.47E-13
45	hsa04672	Intestinal immune network for IgA production	14 of 44	2.41E-13
46	hsa05323	Rheumatoid arthritis	17 of 84	2.62E-13
47	hsa04151	**PI3K-Akt signaling pathway**	29 of 348	5.19E-13
48	hsa05133	Pertussis	16 of 74	5.79E-13
49	hsa04650	Natural killer cell mediated cytotoxicity	19 of 124	6.79E-13
50	hsa05215	Prostate cancer	17 of 97	1.91E-12

Table S11. The proteins of the PPI network involved in the KEGG pathways.

Pathway	Description	#	Proteins involved in the KEGG pathway
**hsa04659**	Th17 cell differentiation	39	CD3E,CD4,CHUK,FOXP3,HLA-DMA,HLA-DMB,HLA-DQA1, HLA-DQA2,HLA-DRA,HLA-DRB1,HLA-DRB5,IFNG,IKBKB, IKBKG,IL12RB1,IL1R1,IL2,IL23A,IL23R,IL2RA,IL2RB, IL2RG,IL6,IL6R,IL6ST,LCK,MAPK14,NFATC2,NFKBIA, PRKCQ,RELA,RUNX1,STAT3,STAT5A,STAT5B,TGFB1, TGFBR1,TGFBR2,ZAP70 (blue)
**hsa04940**	Type I diabetes mellitus	26	CD80,CD86,CPE,GAD1,GAD2,GZMB,HLA-A,HLA-B, HLA-DMA,HLA-DMB,HLA-DQA1,HLA-DQA2,HLA-DRA, HLA-DRB1,HLA-DRB5,HLA-G,ICA1,IFNG,IL12A,IL12B,IL2, INS,LTA,PTPRN,PTPRN2,TNF (red)
**hsa04658**	Th1 and Th2 cell differentiation	30	CD3E,CD4,CHUK,HLA-DMA,HLA-DMB,HLA-DQA1, HLA-DQA2,HLA-DRA,HLA-DRB1,HLA-DRB5,IFNG,IKBKB, IKBKG,IL12A,IL12B,IL12RB1,IL12RB2,IL2,IL2RA,IL2RB, IL2RG,LCK,MAPK14,NFATC2,NFKBIA,PRKCQ,RELA, STAT5A,STAT5B,ZAP70 (green)
**hsa04064**	NF-kappa B signaling pathway	30	BIRC2,BIRC3,BTK,CCL4,CHUK,IKBKB,IKBKG,IL1R1,LCK, LTA,LTBR,LYN,MAP3K7,NFKBIA,PARP1,PRKCQ,RELA, RIPK1,SYK,TAB1,TAB2,TNF,TNFAIP3,TNFRSF1A,TRADD, TRAF2,TRAF6,UBE2I,XIAP,ZAP70 (yellow)
**hsa04210**	Apoptosis	31	APAF1,BIRC2,BIRC3,CASP10,CASP2,CASP3,CASP6,CASP7, CASP8,CASP9,CHUK,DAXX,DFFA,FADD,GZMB,IKBKB, IKBKG,ITPR1,ITPR3,NFKBIA,PARP1,PIK3R1,RELA,RIPK1, SPTAN1,TNF,TNFRSF1A,TNFSF10,TRADD,TRAF2,XIAP (pink)
**hsa04668**	TNF signaling pathway	28	BIRC2,BIRC3,CASP10,CASP3,CASP7,CASP8,CCL5,CHUK, FADD,IKBKB,IKBKG,IL18R1,IL6,LTA,MAP3K7,MAPK14, NFKBIA,PIK3R1,RELA,RIPK1,TAB1,TAB2,TNF,TNFAIP3, TNFRSF1A,TNFRSF1B,TRADD,TRAF2 (orange)
**hsa04060**	Cytokine-cytokine receptor interaction	34	CCL11,CCL4,CCL5,CCR5,CXCR4,EGFR,IFNG,IL12A, IL12B,IL12RB1,IL12RB2,IL18,IL18R1,IL18RAP,IL1R1, IL1R2,IL2,IL23A,IL23R,IL2RA,IL2RB,IL2RG,IL6,IL6R, IL6ST,LTA,LTBR,TGFB1,TGFBR1,TGFBR2,TNF, TNFRSF1A,TNFRSF1B,TNFSF10
**hsa04660**	T cell receptor signaling pathway	23	CBLB,CD3E,CD4,CHUK,CTLA4,FYN,IFNG,IKBKB, IKBKG,IL2,LCK,MAP3K7,MAPK14,NCK1,NFATC2, NFKBIA,PAK2,PIK3R1,PRKCQ,RELA,TNF,VAV1,ZAP70
**hsa04620**	Toll-like receptor	22	CASP8,CCL4,CCL5,CD80,CD86,CHUK,FADD,IKBKB,IKBKG,

	signaling pathway		IL12A,IL12B,IL6,MAP3K7,MAPK14,NFKBIA,PIK3R1,RELA,RIPK1,TAB1,TAB2,TNF,TRAF6
**hsa04010**	MAPK signaling pathway	32	ARRB1,CASP3,CHUK,CRK,CRKL,DAXX,EGFR,IGF1,IGF1R,IGF2,IKBKB,IKBKG,IL1R1,INS,INSR,MAP3K7,MAPK14,MAPT,PAK2,RELA,STK3,STK4,TAB1,TAB2,TGFB1,TGFBR1,TGFBR2,TNF,TNFRSF1A,TRADD,TRAF2,TRAF6
**hsa04910**	Insulin signaling pathway	22	CALM1,CBL,CBLB,CRK,CRKL,G6PC2,GCK,IKBKB,INS,INSR,IRS1,IRS2,IRS4,PHKA1,PHKA2,PHKB,PHKG2,PIK3R1,PTPN1,RPS6KB1,SHC1,SLC2A4
**hsa04630**	Jak-STAT signaling pathway	22	CDKN1A,CREBBP,EGFR,IFNG,IL12A,IL12B,IL12RB1,IL12RB2,IL2,IL23A,IL23R,IL2RA,IL2RB,IL2RG,IL6,IL6R,IL6ST,PIK3R1,PTPN11,STAT3,STAT5A,STAT5B
**hsa04151**	PI3K-Akt signaling pathway	29	CASP9,CDKN1A,CHUK,EGFR,G6PC2,IGF1,IGF1R,IGF2,IKBKB,IKBKG,IL2,IL2RA,IL2RB,IL2RG,IL6,IL6R,INS,INSR,IRS1,ITGAV,ITGB6,ITGB8,PIK3R1,PPP2R5A,RELA,RPS6KB1,SYK,THBS1,YWHAE

Table S12. The miRNAs associated with T1D.

#	miRNA	PMID	Description	Causality
1	hsa-let-7g	30259606	Temporal dynamics of serum let-7g expression mirror the decline of residual beta-cell function in longitudinal observation of children with type 1 diabetes.	NO
2	hsa-mir-100 -5p	28986402	Regarding circulating miRNAs, 11 were consistently dysregulated in T1DM patients compared to controls: miR-21-5p, miR-24-3p, miR-100-5p, miR-146a-5p, miR-148a-3p, miR-150-5p, miR-181a-5p, miR-210-5p, miR-342-3p, miR-375 and miR-1275.	NO
3	hsa-mir-103a -3p	29679626	miR-103a-3p, miR-155-5p, miR-200a-3p, and miR-210-3p were confirmed as being upregulated in recently-diagnosed T1DM patients compared with controls or patients with ≥5 years of diagnosis	NO
4	hsa-mir-1275	28986402	Regarding circulating miRNAs, 11 were consistently dysregulated in T1DM patients compared to controls: miR-21-5p, miR-24-3p, miR-100-5p, miR-146a-5p, miR-148a-3p, miR-150-5p, miR-181a-5p, miR-210-5p, miR-342-3p, miR-375 and miR-1275.	NO
5	hsa-mir-146	24796653	Decreased miR-146 expression in peripheral blood mononuclear cells is correlated with ongoing islet autoimmunity in type 1 diabetes patients.	NO
6	hsa-mir-146a -5p	28986402	Regarding circulating miRNAs, 11 were consistently dysregulated in T1DM patients compared to controls: miR-21-5p, miR-24-3p, miR-100-5p, miR-146a-5p, miR-148a-3p, miR-150-5p, miR-181a-5p, miR-210-5p, miR-342-3p, miR-375 and miR-1275.	NO
7	hsa-mir-146a -5p	30260972	miR-146a-5p could play a role in type 1 diabetes development.	NO
8	hsa-mir-148a -3p	28986402	Regarding circulating miRNAs, 11 were consistently dysregulated in T1DM patients compared to controls: miR-21-5p, miR-24-3p, miR-100-5p, miR-146a-5p, miR-148a-3p, miR-150-5p, miR-181a-5p, miR-210-5p, miR-342-3p, miR-375 and miR-1275.	NO
9	**hsa-mir-149 -5p**	**27737950**	**MicroRNAs miR-23a-3p, miR-23b-3p, and miR-149-5p regulate the expression of proapoptotic BH3-only proteins DP5 and PUMA in human pancreatic β-cells.**	**YES**
10	hsa-mir-150 -5p	28986402	Regarding circulating miRNAs, 11 were consistently dysregulated in T1DM patients compared to controls: miR-21-5p, miR-24-3p, miR-100-5p, miR-146a-5p, miR-148a-3p, miR-150-5p, miR-181a-5p, miR-210-5p, miR-342-3p, miR-375 and miR-1275.	NO
11	hsa-mir-155 -5p	29679626	miR-103a-3p, miR-155-5p, miR-200a-3p, and miR-210-3p were confirmed as being upregulated in recently-diagnosed T1DM patients compared with controls or patients with ≥5 years of diagnosis.	NO
12	hsa-mir-181a -5p	28986402	Regarding circulating miRNAs, 11 were consistently dysregulated in T1DM patients compared to controls: miR-21-5p, miR-24-3p, miR-100-5p, miR-146a-5p, miR-148a-3p, miR-150-5p, miR-181a-5p, miR-210-5p, miR-342-3p, miR-375 and miR-1275.	NO
13	hsa-mir-181a	26892629	miRNA-181a expression was significantly higher in diabetic children and adolescents.	NO
14	**hsa-mir-192**	**30186503**	**miR-192 is upregulated in T1DM, regulates pancreatic β-cell development and inhibits insulin secretion through suppressing GLP-1 expression.**	**YES**
15	hsa-mir-200	26244930	These data strongly associate miR200-mediated	NO

			downregulation of the DNA damage checkpoint proteins with propensity for developing microvascular complications of T1D.	
16	hsa-mir-200a -3p	29679626	miR-103a-3p, miR-155-5p, miR-200a-3p, and miR-210-3p were confirmed as being upregulated in recently-diagnosed T1DM patients compared with controls or patients with ≥5 years of diagnosis.	NO
17	hsa-mir-20a	30194557	Increased expression of microRNAs, miR-20a and miR-326 in PBMCs of patients with type 1 diabetes.	NO
18	hsa-mir-21 -5p	28986402	Regarding circulating miRNAs, 11 were consistently dysregulated in T1DM patients compared to controls: miR-21-5p, miR-24-3p, miR-100-5p, miR-146a-5p, miR-148a-3p, miR-150-5p, miR-181a-5p, miR-210-5p, miR-342-3p, miR-375 and miR-1275.	NO
**19**	**hsa-mir-21**	**28280903**	**MicroRNA 21 targets BCL2 mRNA to increase apoptosis in rat and human beta cells.**	**YES**
20	hsa-mir-21	23506112	Though the exact roles of miR-21 in autoimmune diseases have not been fully elucidated, targeting miR-21 may serve as a promising therapy.	NO
21	hsa-mir-210 -3p	29679626	miR-103a-3p, miR-155-5p, miR-200a-3p, and miR-210-3p were confirmed as being upregulated in recently-diagnosed T1DM patients compared with controls or patients with ≥5 years of diagnosis.	NO
22	hsa-mir-210 -5p	28986402	Regarding circulating miRNAs, 11 were consistently dysregulated in T1DM patients compared to controls: miR-21-5p, miR-24-3p, miR-100-5p, miR-146a-5p, miR-148a-3p, miR-150-5p, miR-181a-5p, miR-210-5p, miR-342-3p, miR-375 and miR-1275.	NO
**23**	**hsa-mir-23a -3p**	**27737950**	**MicroRNAs miR-23a-3p, miR-23b-3p, and miR-149-5p regulate the expression of proapoptotic BH3-only proteins DP5 and PUMA in human pancreatic β-cells.**	**YES**
**24**	**hsa-mir-23b -3p**	**27737950**	**MicroRNAs miR-23a-3p, miR-23b-3p, and miR-149-5p Regulate the Expression of Proapoptotic BH3-Only Proteins DP5 and PUMA in Human Pancreatic β-Cells.**	**YES**
25	hsa-mir-24 -3p	28986402	Regarding circulating miRNAs, 11 were consistently dysregulated in T1DM patients compared to controls: miR-21-5p, miR-24-3p, miR-100-5p, miR-146a-5p, miR-148a-3p, miR-150-5p, miR-181a-5p, miR-210-5p, miR-342-3p, miR-375 and miR-1275.	NO
26	hsa-mir-326	22069274	Increased expression of microRNA miR-326 in type 1 diabetic patients with ongoing islet autoimmunity.	NO
27	hsa-mir-326	30194557	Increased expression of microRNAs, miR-20a and miR-326 in PBMCs of patients with type 1 diabetes.	NO
28	hsa-mir-342 -3p	28986402	Regarding circulating miRNAs, 11 were consistently dysregulated in T1DM patients compared to controls: miR-21-5p, miR-24-3p, miR-100-5p, miR-146a-5p, miR-148a-3p, miR-150-5p, miR-181a-5p, miR-210-5p, miR-342-3p, miR-375 and miR-1275	NO
29	hsa-mir-34a	28185128	Effects of TRPM7/miR-34a Gene Silencing on Spatial Cognitive Function and Hippocampal Neurogenesis in Mice with Type 1 Diabetes Mellitus.	NO
30	hsa-mir-375	28986402	Regarding circulating miRNAs, 11 were consistently dysregulated in T1DM patients compared to controls: miR-21-5p, miR-24-3p, miR-100-5p, miR-146a-5p, miR-148a-3p, miR-150-5p, miR-181a-5p, miR-210-5p, miR-342-3p, miR-375 and miR-1275	NO
31	hsa-mir-375	30150203	Several types of miRNAs are playing important roles	NO

			in type 1 diabetes mellitus including miR-375 and miR-375 with intolerance to glucose and decreased beta cells account due to impaired proliferation	
32	hsa-mir-487a -3p	29859273	miR-487a-3p upregulated in type 1 diabetes targets CTLA4 and FOXO3.	NO

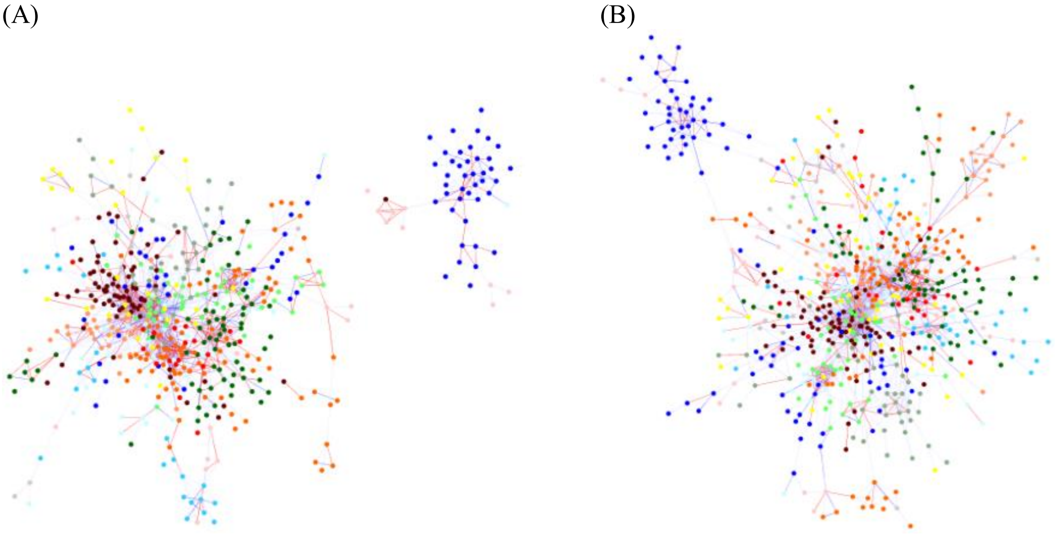

ICD9	Disease Chapter	Color	ICD9	Disease Chapter	Color
001-139	infectious and parasitic diseases		390-459	diseases of the circulatory system	
140-239	neoplasms		460-519	diseases of the respiratory system	
240-279	endocrine, nutritional and metabolic diseases, and immunity disorders		520-579	diseases of the digestive system	
250	diabetes mellitus		580-629	diseases of the genitourinary system	
280-289	diseases of the blood and blood-forming organs		680-709	diseases of the skin and subcutaneous tissue	
290-319	mental disorders		710-739	diseases of the musculoskeletal system and connective tissue	
320-389	diseases of the nervous system and sense organs		740-759	congenital anomalies	

Figure S1. The PDNs of control group for (A) Male and (B) Female inpatients.

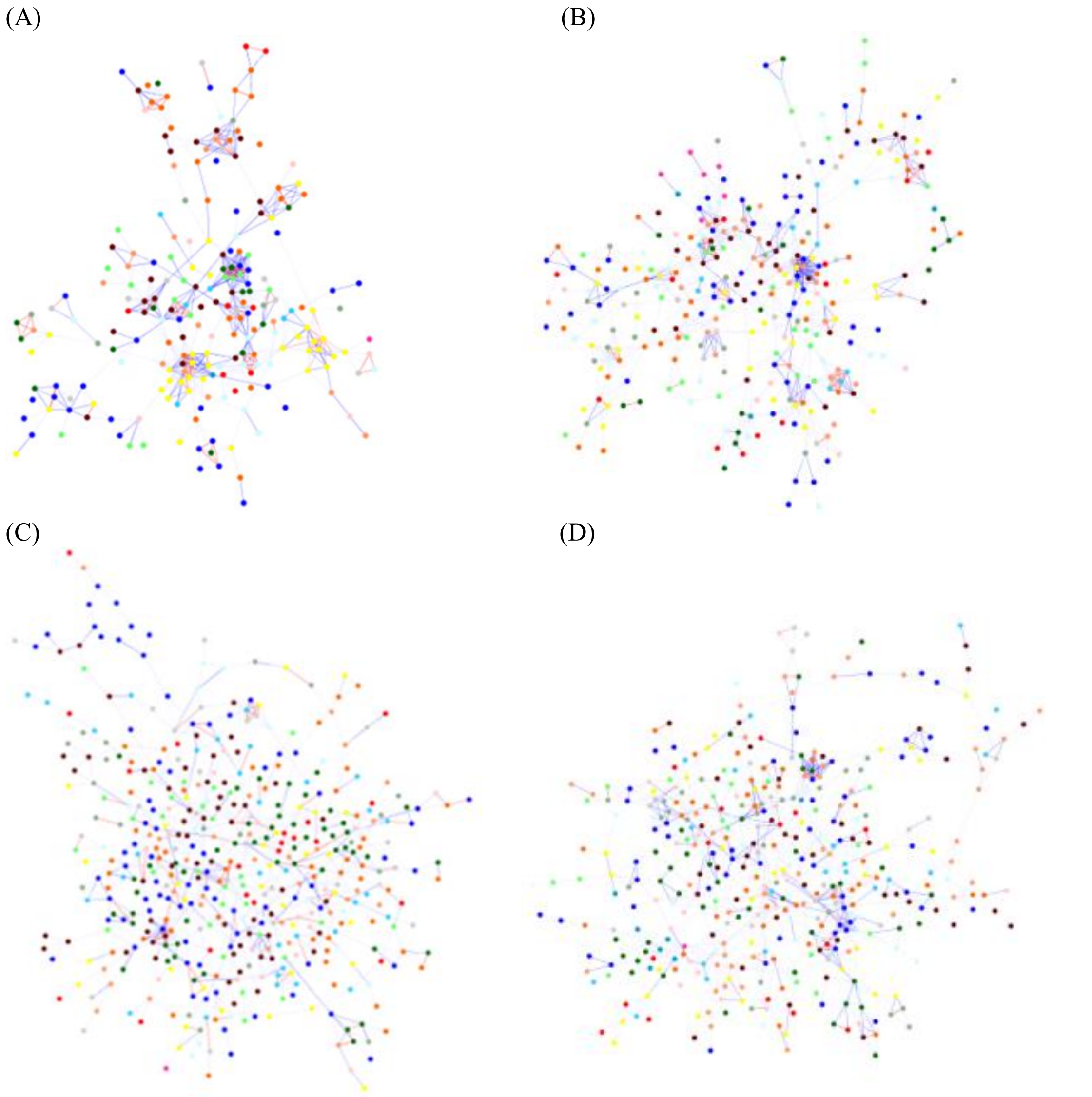

ICD9	Disease Chapter	Color	ICD9	Disease Chapter	Color
001-139	infectious and parasitic diseases		390-459	diseases of the circulatory system	
140-239	neoplasms		460-519	diseases of the respiratory system	
240-279	endocrine, nutritional and metabolic diseases, and immunity disorders		520-579	diseases of the digestive system	
250	diabetes mellitus		580-629	diseases of the genitourinary system	
280-289	diseases of the blood and blood-forming organs		680-709	diseases of the skin and subcutaneous tissue	
290-319	mental disorders		710-739	diseases of the musculoskeletal system and connective tissue	
320-389	diseases of the nervous system and sense organs		740-759	congenital anomalies	

Figure S2. The PDNs of T1D for (A) 1-20 male, (B) 1-20 female, (C) 40-60 male, and (D) 40-60 female inpatients.

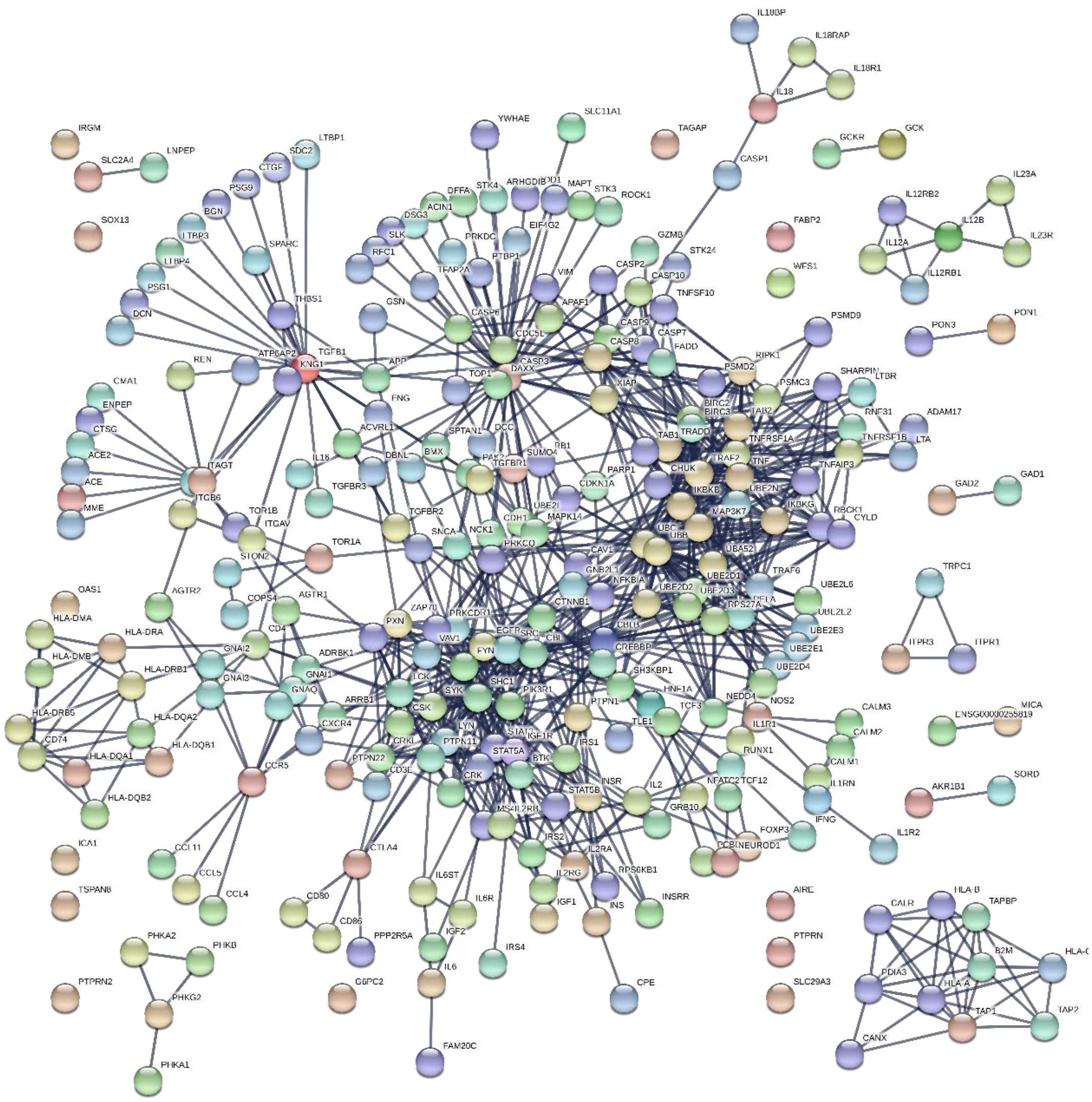

# of nodes	# of links	Average node degree	PPI enrichment *p*-value
278	919	6.61	< 1.0e-16

Figure S3. The PPI network of T1D.

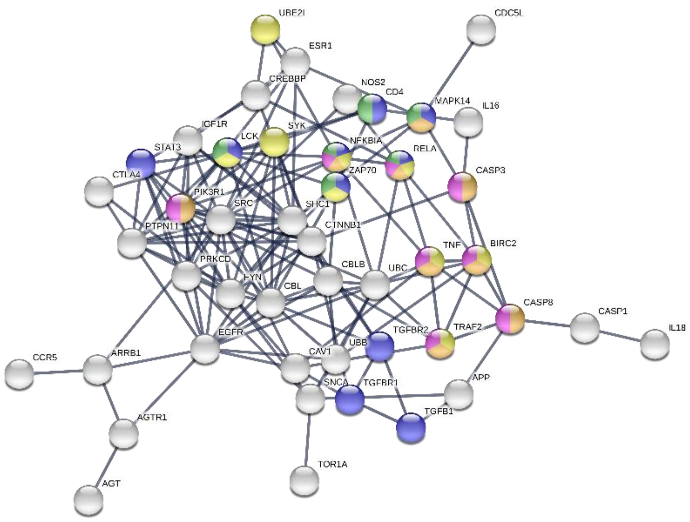

Figure S4. The backbone network.

The nodes involved in Th17 cell differentiation (hsa04659), Th1 and Th2 cell differentiation (hsa04658), NF-kappa B signaling pathway (hsa04064), apoptosis (hsa04210), and TNF signaling pathway (hsa04668) were colored in blue, green, yellow, pink, and orange, respectively.
